# Limited clonal relatedness between gut IgA plasma cells and memory B cells after oral immunization

**DOI:** 10.1038/ncomms12698

**Published:** 2016-09-06

**Authors:** Mats Bemark, Helena Hazanov, Anneli Strömberg, Rathan Komban, Joel Holmqvist, Sofia Köster, Johan Mattsson, Per Sikora, Ramit Mehr, Nils Y. Lycke

**Affiliations:** 1 Mucosal Immunobiology and Vaccine Research Center and the Department of Microbiology and Immunology, Institute of Biomedicine, University of Gothenburg, S-405-30 Gothenburg, Sweden; 2 Department of Clinical Immunology and Transfusion Medicine, Sahlgrenska University Hospital, S-405-30 Gothenburg, Sweden; 3 Computational Immunology Lab, The Mina and Everard Goodman Faculty of Life Sciences, Bar-Ilan University, 5290002 Ramat-Gan, Israel; 4 Department of Laboratory Medicine, Sahlgrenska University Hospital, S-405-30 Gothenburg, Sweden

## Abstract

Understanding how memory B cells are induced and relate to long-lived plasma cells is important for vaccine development. Immunity to oral vaccines has been considered short-lived because of a poor ability to develop IgA B-cell memory. Here we demonstrate that long-lived mucosal IgA memory is readily achieved by oral but not systemic immunization in mouse models with NP hapten conjugated with cholera toxin and transfer of B1-8^high^/GFP^+^ NP-specific B cells. Unexpectedly, memory B cells are poorly related to long-lived plasma cells and less affinity-matured. They are α4β7-integrin^+^CD73^+^PD-L2^+^CD80^+^ and at systemic sites mostly IgM^+^, while 80% are IgA^+^ in Peyer's patches. On reactivation, most memory B cells in Peyer's patches are GL7^−^, but expand in germinal centres and acquire higher affinity and more mutations, demonstrating strong clonal selection. CCR9 expression is found only in Peyer's patches and appears critical for gut homing. Thus, gut mucosal memory possesses unique features not seen after systemic immunization.

## Abstract (web summary)

The presence of long-lived memory IgA-producing B cells in the intestine has been difficult to document. Here the authors show that these cells can be induced by oral but not systemic immunization in mice, and characterize unique features that distinguish them from IgA plasma cells.

## Graphical abstract

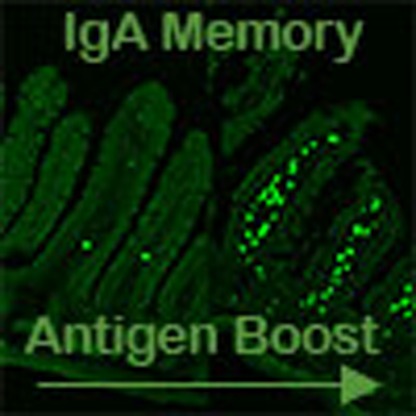

Conflicting reports on the ability of the mucosal immune system to generate long-term IgA antibody production and memory B cells have recently been published. On one hand, studies on enteric infectious diseases, such as cholera and rotavirus infections, have clearly documented strong IgA memory development^1^,^2^. On the other hand, protection against infection after mucosal vaccination has been considered short-lived and studies of bacterial colonization in germ-free mice have indicated that specific IgA B-cell memory fails to develop^3^,^4^,^5^. Yet, investigations of IgA V region gene sequences in young and adult mice have revealed a progressive accumulation of somatic hypermutations with age, suggesting the buildup of a memory B-cell pool^6^,^7^. In addition, IgA production in the gut lamina propria (LP) of individual mice exhibited essentially an identical repertoire and clonality to that seen before depletion of gut IgA plasma cells with Bortezomib, which suggests the presence of memory B cells in the gut immune system^6^,^7^. Hence, whether mucosal long-term IgA memory should be considered poorly developed compared with systemic long-term memory is, from an evolutionary perspective, an unresolved question and an issue of current debate.

Whereas our group and others have demonstrated long-lived IgA plasma cells in the gut LP and memory B cells in secondary lymphoid tissues after oral immunizations in mice, little detailed information is available as to the regulatory mechanisms, physical localization and clonal relationships of these cells^8^,^9^,^10^,^11^,^12^. An oral booster immunization with cholera toxin (CT) 24 months after priming elicited a very strong gut antitoxin IgA memory response and, similarly, oral rotavirus immunization stimulated long-term memory that protected against infection through production of local IgA antibodies^10^,^12^. Whereas the latter is an example of what appears to be T-cell- and germinal centre (GC)-independent IgA-mediated protection, the antitoxin IgA response is clearly T-cell and GC dependent^13^,^14^,^15^. Of note, a GC-independent pathway for B-cell memory development has recently been demonstrated, but unlike GC-dependent memory B cells, these cells exhibited few IgH V gene mutations^16^. Thus, to what extent GC reactions are critical for B-cell memory development in the gut is incompletely understood. Furthermore, whether such cells are isotype-switched memory B cells or represent persistent IgM memory B cells, as has been observed after rotavirus infections in humans, is presently attracting attention^2^. GC-dependent IgM memory B cells have been found to carry a high frequency of somatic hypermutations and effectively establish secondary GC reactions, and undergo isotype switching on reactivation^17^,^18^. In contrast, switched memory B cells rapidly differentiated into antibody-forming cells (AFCs) but did not form GC. Notably, human IgM memory B cells can undergo isotype switching on reactivation as shown with rotavirus both *ex vivo* and *in vivo*^2^. However, whether isotype switching prevents memory B cells from participating in secondary GC reactions was recently questioned by Shlomchik and co-workers, who found that, irrespective of isotype, cell-intrinsic properties, associated with the expression of the memory markers CD80 and PD-L2, were critical for the ability to engage in secondary GC reactions^19^. They found that double-positive memory B cells became AFCs, but did not form GC, while double-negative cells established secondary GC. To what extent these findings apply to mucosal memory B cells has not been investigated.

Gut IgA-producing plasma cells are constantly generated through class switch recombination of activated B cells in the Peyer's patches (PPs), which are the main sites for both T-cell-dependent and T-cell-independent IgA responses^15^,^20^. Gut IgA production is critically dependent on the microbiota and germ-free mice fail to develop significant IgA responses^21^,^22^. The exact reason for this is not completely understood, but the unique microenvironment in the PP inductive sites and/or the formation of an IgA plasma cell survival niche in the LP are both possible contributing factors^23^. Hence, gut mucosal memory B-cell development can be dependent on the presence and composition of the microbiota. The involvement of Th17 and Foxp3^+^ regulatory T cells in the generation of gut IgA immunity also make the PP and LP stand out as different and quite unique environments in relation to the regulatory elements that are involved in systemic memory B-cell responses^24^,^25^. Importantly, because of the microbiota, we have a consistent presence of GC in the PPs and, therefore, the PP is a potential memory development site for both T-cell-dependent and -independent memory B cells^26^.

Previous studies in humans and mice have documented long-lived antigen-specific IgA plasma cells in the gut LP up to 6 months after oral immunization^3^,^27^. Long-lived plasma cells also reside in the bone marrow (BM) after oral immunization and produce strong serum IgA as well as IgG antibody levels^28^. Interestingly, little is known about the clonal relationships between IgA and IgG long-lived plasma cells or memory B cells in the various locations. Our earlier studies have indicated the existence of a regulatory dichotomy between mucosal IgA and systemic IgG immunity, as observed in the CD28-deficient mouse^29^. The present study was undertaken to shed light on the regulatory requirements for mucosal memory B-cell development using the hapten (4-hydroxy-3-nitrophenyl)acetyl (NP) conjugated with CT as an oral immunogen in wild-type mice or mice adoptively transferred with B1-8^high^/GFP^+^ NP-specific B cells^30^.

## Results

### Clonally related long-lived plasma cells in the gut and BM

**Figure 1:**
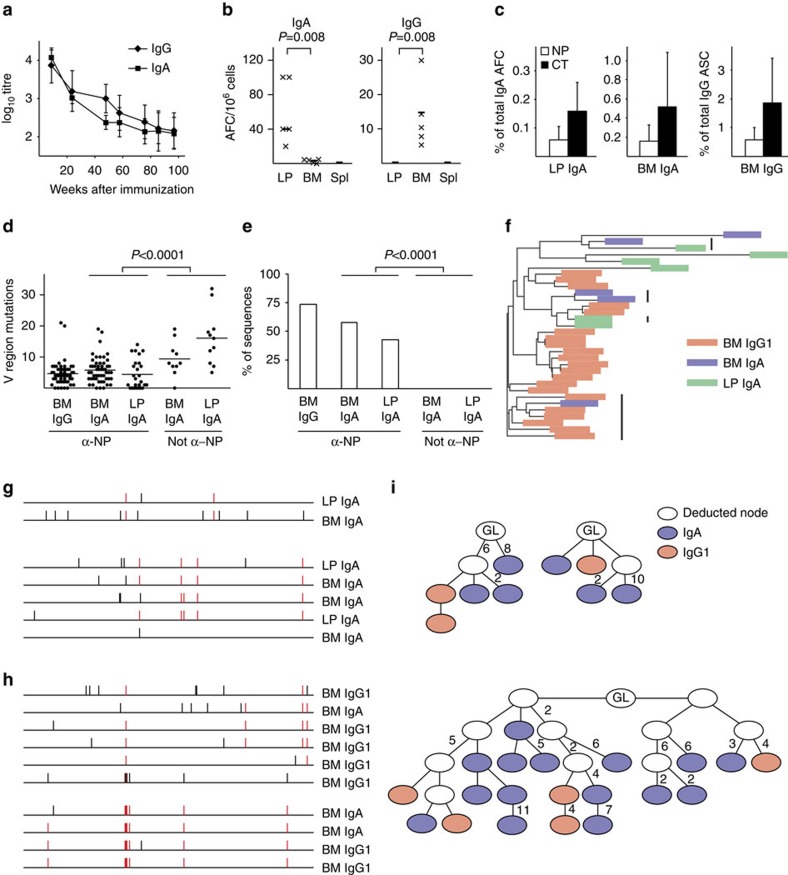
Oral priming immunizations generate clonally related long-lived plasma cells in the gut lamina propria and bone marrow. (**a**) Wild-type C57BL/6 mice were orally primed with three doses of 20 μg NP-CT at 10 days apart and serum anti-NP or anti-CT titres of the IgG (diamonds) or IgA (squares) class were determined by enzyme-linked immunosorbent assay at the indicated time points and shown with s.d. error bars. (**b**,**c**) After 1 year, lymphocytes were isolated from the small intestinal lamina propria (SI LP), bone marrow (BM) or spleen (Spl), and the total number of anti-NP or anti-CT IgA or IgG antibody-forming cells (AFCs)/10^6^ isolated cells were determined by enzyme-linked immunospot. (**b**) The number of NP-specific IgA and IgG AFCs in the different tissues and (**c**) the proportion of NP or CT-specific AFCs of all IgA or IgG AFCs in the LP or BM are shown with s.d. error bars. (**d**–**g**) RNA was prepared from the SI or BM from six mice, and NP-binding heavy chain IgG or IgA genes were cloned and sequenced. The average number of IgH V region mutations in unique sequences (**d**) and the proportion of sequences that carried the affinity-increasing CDR1 W33=>L or CDR3 Y=>G mutations (**e**) were determined in V_H_186.2 gene rearrangements. Mann–Whitney test *P* values are given. The method used to define NP-binding V_H_186.2 gene sequences as opposed to non-NP-binding sequences is described in the Methods section. (**f**) Clustal Omega analysis was used to determine sequence similarities in individual mice. Clones that share CDR3 VDJ rearrangements are marked with black lines. (**g**,**h**) Schematic representation of clones from the SI LP and BM that share IgA V region rearrangements (**g**) or IgA and IgG1 clones from the BM that share V region gene sequences (**h**). Point mutations in the V regions are marked in red if shared with other sequences in the group and black if unique to a single sequence. (**i**) Clonal tree analysis of clonally related NP-binding V_H_186.2 sequences from individual mice identified clones that contain both IgA and IgG1 V region gene sequences. The number of mutations between neighbouring nodes is given next to the connecting edge, where no number is given the edge represents a single mutation. Data from five to six mice in each group in one representative experiment (**a**–**c**) of three giving similar results (pooled data in **d**–**f**).

A thorough investigation of the ability to form and maintain memory B cells and long-lived plasma cells in various locations after oral immunization is still lacking^12^. Therefore, we explored the NP–CT hapten-carrier system, which we previously have used to demonstrate clonal relationships between NP-specific IgA B cells in inductive and effector sites following oral immunization^30^. We found that after three oral immunizations with NP-CT, high antigen-specific serum IgA and IgG titres could be observed for more than 100 weeks, that is, almost lifelong (Fig. 1a; Supplementary Fig. 1a). Interestingly, specific serum titres dropped more rapidly over the first 40–60 weeks with a half-life of 2 months, while after this time they remained relatively stable at a level 30–100-fold lower than the early peak response (Fig. 1a; Supplementary Fig. 1a). The serum antibody titres correlated with the presence of NP- and CT-specific IgA-secreting plasma cells in both the small intestinal (SI) LP and the BM, but not in the spleen. A dichotomy between IgA and IgG plasma cells was evident with IgG plasma cells restricted to the BM and IgA plasma cells found in the SI LP and BM (Fig. 1b; Supplementary Fig. 1b). After 1 year, a notable frequency of specific plasma cells was still maintained in the BM and SI LP with 0.5% and 2% IgG or 0.05% and 0.2% IgA, anti-NP or anti-CT AFCs, respectively, out of total AFCs in these locations (Fig. 1c).

Next, we sequenced (using traditional Sanger sequencing) the V_H_186.2 heavy chain V region gene, encoding the NP-binding antibodies, and found that long-lived IgA and IgG plasma cells exhibited a similar frequency of mutations with an average of five mutations in the V regions, and 50% of the sequenced IgA genes carried high-affinity mutations (Fig. 1d,e). Of note, ∼90% of all NP-binding IgG transcripts were of the IgG1 subclass, reflecting a clear Th2 skewing (IL-4) of the oral NP-CT response (Supplementary Fig. 1c)^31^. An analysis of clonal relationships revealed that IgA and IgG plasma cells shared similar rearranged V regions, and IgA and IgG NP-binding cells in the SI LP and BM were, indeed, clonally related (Fig. 1f–i; Supplementary Figs 1c and 2).

### Oral priming immunizations stimulate mucosal memory B cells

**Figure 2:**
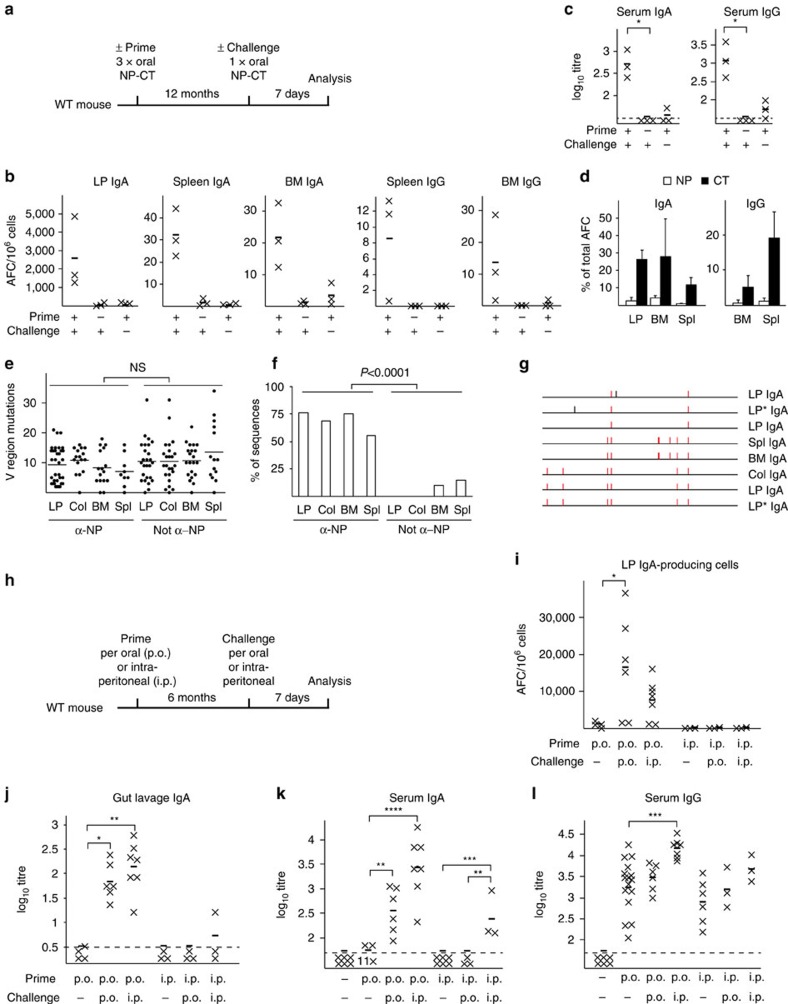
Re-exposure to oral or systemic antigen after 1 year markedly boosts local and systemic immune responses in orally primed memory B cells. (**a**) Wild-type C57BL/6 mice were orally primed with three doses of 20 μg NP-CT at 10 days apart and challenged 1 year later with a single oral challenge dose with NP-CT. (**b**) After 7 days, the numbers of anti-NP IgA (left) or IgG (right) AFCs/10^6^ cells in different tissues were determined by enzyme-linked immunospot in individual primed-only, primed and challenged or age-matched challenged-only mice. (**c**) Serum IgA or IgG anti-NP titres log_10_ titres in individual mice. The dashed line identifies the detection limit for the assay. (**d**) The proportions of anti-NP or anti-CT IgA (left) or IgG (right) AFCs of total AFCs in indicated tissues are shown with s.d. error bars. (**e**–**g**) RNA was prepared from the SI LP, colon (Col), BM or spleen (Spl) from boosted mice, and NP-binding V_H_186.2 genes were cloned and analysed: (**e**) V region mutations, (**f**) the frequency of the affinity-increasing CDR1 W33=>L or CDR3 Y=>G mutations and (**g**) a representation of clones from different tissues that share IgA V region rearrangements, with red indicating shared and black unique mutations. (**h**–**l**) Mice were either orally or intraperitoneally (i.p.) primed with NP-CT or NP-CGG plus CTA1-DD adjuvant, respectively, and then challenged after 6 months with NP-CT orally or i.p., as indicated (**h**). The number of anti-NP AFCs/10^6^ cells in the SI LP in individual mice is shown (**i**). Anti-NP IgA log_10_ titres in gut lavage (**j**) or serum (**k**) or serum anti-NP IgG log_10_ titres (**l**) in individual mice are shown. Data from three to six mice in each group in one representative experiment of three giving similar results (**b**–**d**). Graphs (**e**,**f**,**i**,**i**–**l**) are based on pooled data from three independent experiments. Mann–Whitney test *P* values are given (*0.01≤*P*<0.05, **0.005≤*P*<0.01, ***0.001≤*P*<0.005, ****P≤0.001).

We boosted orally primed mice after 1 year and observed a rapid increase in antigen-specific IgA AFCs in the SI LP and IgA and IgG AFCs in the spleen and BM (Fig. 2a,b; Supplementary Fig. 3a). Specific serum antibodies increased >20-fold within a week (average 1.5 and 1.7 log_10_ titres for IgG and IgA, respectively) after the oral challenge immunization as opposed to the poor titres seen in previously unimmunized age-matched control mice (Fig. 2c; Supplementary Fig. 3b). NP-specific IgA AFCs increased to between 0.5 and 4% of all AFCs, while CT-specific AFCs reached 5–25% of all AFC, in the SI LP and BM (Fig. 2d). Specific IgG AFCs were most frequent in the spleen (Fig. 2d). After an oral challenge immunization responding NP-specific cells carried more mutations (average 10 mutations) compared with those seen in long-lived plasma cells (average five mutations) (Figs 1d, 2e). In addition, an increase from ∼50 to 75% of cells carrying the high-affinity CDR1 mutation was noted (Figs 1e, 2f). Importantly, we found that NP-binding IgA sequences shared clonal origin irrespective of where they were isolated from the SI LP, BM or spleen, arguing in favour of an effective clonal selection process following oral booster immunizations (Fig. 2g; Supplementary Fig. 4). Hence, it appeared that oral priming immunizations effectively promoted the development of antigen-specific memory B cells at both mucosal and systemic sites.

Because many researchers have attempted to immunize systemically to achieve strong mucosal IgA memory responses, we analysed whether systemic priming immunizations also could stimulate mucosal anti-NP or anti-CT memory B cells. To this end, we immunized mice with an optimal dose of NP-CGG plus CTA1-DD adjuvant intraperitoneally (i.p.) and after 6 months the mice were challenged orally (p.o.) or i.p. with NP-CT (Fig. 2h)^32^. We found that i.p. priming followed by a p.o. boost failed to stimulate anti-NP and anti-CT IgA AFCs in the SI LP or serum IgA responses (Fig. 2i–k; Supplementary Fig. 3c–e). By contrast, it was clear that an i.p. boost to p.o. NP-CT primed mice effectively triggered strong NP- and CT-specific gut and serum IgA responses, comparable even to those seen after a p.o. boost (Fig. 2i–k; Supplementary Fig. 3c–e). Systemic priming immunizations were effective at stimulating systemic anti-NP and anti-CT IgG memory; however, this was also achieved by p.o. priming immunizations with NP-CT (Fig. 2l; Supplementary Fig. 3d,f). Thus, p.o. priming immunizations were effective at generating memory B cells that could provide broad mucosal IgA and systemic IgG antibody responses on systemic or oral antigen re-challenge. Only oral priming imprinted gut homing properties in memory B cells, while systemic priming failed to do so.

### Long-lived gut memory B cells reside in the follicles

**Figure 3:**
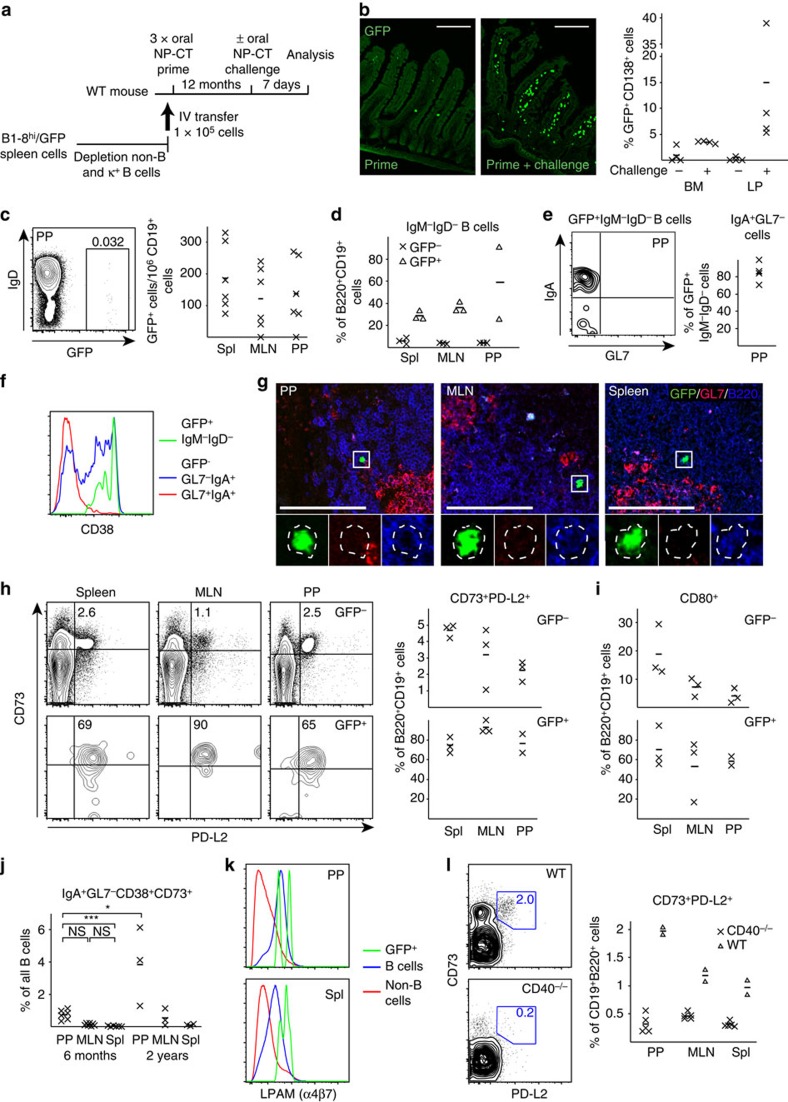
Orally primed memory B cells express CD80, CD73 and PD-L2 and reside in B-cell follicles in mucosal as well as systemic lymphoid tissues. (**a**) An adoptive transfer model with GFP^+^ NP-specific B cells was employed for monitoring memory B-cell development after oral immunization. Wild-type C57BL/6 mice were injected with 100,000 GFP^+^ λ-expressing NP-specific splenic B cells prepared from B1-8^high^/GFP mice, as described in the Methods section. (**b**) One year after oral priming immunizations with NP-CT (left) an oral booster immunization was administered and 7 days later (right) sections of gut LP were analysed for the presence of GFP^+^ NP-specific plasma cells by confocal microscopy. Flow cytometry analysis of the mean (−) and individual (*x*) frequency of GFP^+^CD138^+^ plasma cells in single-cell suspensions from the BM or SI LP. (**c**) Before the booster immunization, we determined the frequency of memory GFP^+^ NP-specific CD138^−^B220^+^CD19^+^ cells/10^6^ total B cells in different tissues (spleen (Spl), MLN or PP) of six mice analysed individually by flow cytometry. (**d**) The proportion of GFP^+^ and GFP^−^ CD138^−^B220^+^CD19^+^ B cells that had an IgM^−^IgD^−^ isotype-switched phenotype in the indicated tissues. (**e**) The percentage of GFP^+^IgM^−^IgD^−^ isotype-switched memory B cells from PP that expressed IgA in four individual mice. (**f**) Expression of CD38 on GFP^+^ memory B cells and GL7^+^ and GL7^−^ IgA-expressing GFP^−^ B cells in PP. (**g**, see also Supplementary Fig. 5b) Representative frozen sections analysed by confocal microscopy for GFP^+^ memory B cells (green) in the PP, MLN and Spl with germinal centres labelled with anti-GL7-eFlour 660 (red) and B-cell follicles with anti-B220-biotin/Streptavidin-Alexa Flour 594 (blue). Below the main panels are channel-separated close-ups to demonstrate that the GFP^+^ cells are B220^+^, but do not express GL7. (**h**) Flow cytometry analysis for CD73 and PD-L2 expression on CD138^−^B220^+^CD19^+^ GFP^+^ memory B cells isolated from indicated tissues. For comparisons, GFP^−^ B cells are shown. The percentage of CD73^+^PD-L2^+^ memory B cells in three individual memory mice is depicted (right). (**i**) The frequency of CD80-expressing GFP^+^ memory B cells. (**j**) The percentage of B cells from the PP, MLN or Spl with a memory IgA^+^IgM^−^IgD^−^CD73^+^CD38^+^GL7^−^ phenotype in 6 months (left) or 2-year-old (right) unimmunized mice. (**k**) Expression of the gut homing α4β7 receptor (LPAM integrin) in GFP^+^ memory B cells, as opposed to that found in GFP^−^ B cells or non-B cells in the PP and Spl. (**l**) Lack of CD73^+^PD-L2^+^-expressing CD19^+^ B cells from the PP, MLN or Spl in CD40^−/−^ mice. Representative data from groups of two to six mice and two to four independent experiments giving similar results. Mann–Whitney test and *P* values are given (not significant (NS) *P*>0.05, *0.01≤*P*<0.05, **0.005≤*P*<0.01, ***0.001≤*P*<0.005).

To identify the localization and phenotype of antigen-specific memory B cells formed after oral priming immunizations, we used an adoptive transfer model with B1-8^high^/GFP^+^ NP-specific B cells, as previously described^30^. Naive IgD^high^ GFP-expressing NP-specific B cells were adoptively transferred into recipient mice, which were then given three oral priming immunizations with NP-CT (Fig. 3a). One year after priming, these mice exhibited a robust memory response following an oral challenge immunization, resulting in large numbers of GFP^+^ plasma cells detected in the SI LP and BM (Fig. 3b). After oral priming immunizations, the average frequencies of IgD^−^ GFP-expressing B220^+^CD19^+^CD138^−^ memory B cells in various tissues were assessed (gating strategy in Supplementary Fig. 5a). We found similar levels of GFP^+^ NP memory B cells in the spleen, mesenteric lymph nodes (MLN) and PP—on average 190, 120 and 125 per 10^6^ B cells, respectively (Fig. 3c). Interestingly, whereas class-switched NP memory B cells dominated in the PP and 80% of these expressed IgA, in the spleen and MLN a majority expressed IgM, that is, only 20–40% expressed IgG and very few were IgA class-switched cells (Fig. 3d,e). In the PP and other organs, a majority of the GFP^+^ B cells had a resting memory GL7^−^CD38^+^ phenotype, distinctly different from activated B cells in the GC (GL7^+^CD38^−^; Fig. 3e,f; Supplementary Fig. 5b). Memory B cells were B220^+^GL7^−^CD138^−^ and they were found in the follicles in all three organs, often close to GL7^+^ GC areas (Fig. 3g; Supplementary Fig. 5c). Flow cytometry analysis revealed that GFP^+^ memory B220^+^CD19^+^CD138^−^B cells expressed the memory markers CD73, PD-L2 and most also expressed CD80 (refs 33, 34) (Fig. 3h,i; Supplementary Fig. 5d). In fact, a substantial fraction of all B cells (that is, GFP^−^B220^+^ cells) expressed CD73 and PD-L2 (2–5%) in the PP, MLN and spleen in aged mice, supporting the view that all these tissues accumulate memory B cells over time (Fig. 3h–j). IgA-expressing memory B220^+^CD138^−^ B cells dominated in the PP but not in other organs (Fig. 3j; Supplementary Fig. 5e). Importantly, NP-specific GFP^+^ memory B cells isolated from either the PP or spleen expressed significant levels of the gut homing receptor α4β7 (Fig. 3k). By contrast, when i.p. priming immunizations were given memory B cells in the spleen or peripheral lymph nodes did not express the gut homing receptor α4β7 (Supplementary Fig. 5f). Noteworthy, insignificant levels of B cells with a CD73^+^PD-L2^+^ memory phenotype could be found in CD40-deficient mice (lacking GC completely), indicating that most CD73^+^ B cells emanate from GC reactions in normal mice under homeostatic conditions (Fig. 3l).

**Figure 4:**
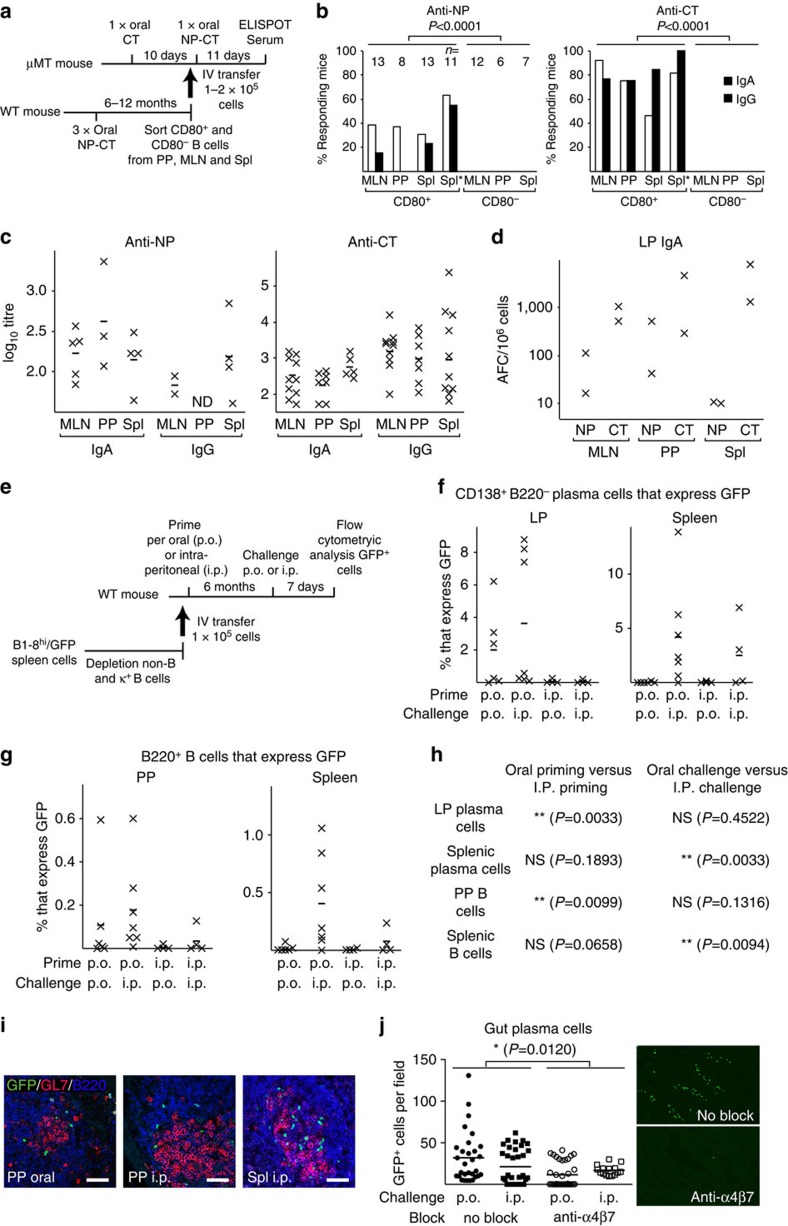
Re-circulating CD80^+^ memory B cells formed after oral immunization generate α4b7-dependent gut IgA responses. (**a**) Wild-type C57BL/6 mice were orally primed with three doses of 20 μg NP-CT at 10 days apart and 6–12 months later CD80^+^ and CD80^−^ B cells from the Spl, MLN or PP were sorted using a FACSAria and injected intravenously into μMT recipient mice that had been orally immunized once with 10 μg of CT 10 days earlier. One day after cell transfer μMT mice were orally immunized with 20 μg NP-CT and the anti-NP and anti-CT responses in serum or SI LP (AFC) were monitored. (**b**) Depicts the frequency of responding mice based on detection of anti-NP (left) or anti-CT (right) IgA or IgG serum antibody responses (as depicted in **c**) after transfer of CD80^+^ or CD80^−^ B cells using 1 × 10^5^ cells from MLN and PP and 2–10 × 10^5^ from the spleen (Spl). The number of recipient mice in each group (*n*=) is given in the left panel for NP responses. (**c**) Specific log_10_ antibody titres in individual mice that responded to an oral challenge immunization (ND, not detectable). (**d**) Small intestinal LP anti-NP or anti-CT IgA responses given as AFCs/10^6^ cells in μMT mice injected with CD80^+^ memory B cells from indicated tissues. The data presented in **a**–**d** are based on the data pooled from four independent experiments giving similar results. (**e**) Mice adoptively transferred with NP-specific GFP-expressing B cells were orally or i.p. primed with NP-CT and 6 months later a challenge dose with NP-CT was given orally or i.p. (**f**–**i**) The percentage of GFP^+^ CD138^+^/all plasma cells in the SI LP and spleen of responding mice (**f**) and GFP^+^ B220^+^/all B cells in the PP and spleen in these mice (**g**). (**h**) A statistical analysis of the impact of p.o. versus i.p. priming or p.o. versus i.p. challenge immunizations for strong IgA responses (explained in Supplementary Fig. 6b,c). (**i**) Tissue sections of the PP and spleen showing GC location of responding of GFP^+^ memory B cells after challenge. (**j**) Orally primed mice were treated with α4β7-blocking DATK32 Mab before and during p.o. or i.p. challenge immunizations with NP-CT and the number of GFP^+^ plasma cells in SI LP sections were determined (explained in Supplementary Fig. 6a). The Mann–Whitney nonparametric test was used for statistical analysis of GFP^+^ gut homing plasma cells in the presence or absence of α4β7 Mab blockade (*P*=0.0120). Results in **e**–**j** are based on the pooled data from three independent experiments.

To be able to assess the ability of memory B-cell clones to expand, we transferred fluorescence-activated cell sorting (FACS)-sorted CD80^+^ and CD80^−^ B cells into B-cell deficient μMT mice that had been pre-immunized with CT to secure adequate T-cell help (Fig. 4a). After a single oral immunization with NP-CT in mice that had received between 75.000 and 100.000 sorted CD80^+^ memory B cells, 35–60% of the mice responded to NP, while 75–100% of the mice responded to CT (Fig. 4b). With a frequency of 100 anti-NP memory B cells/10^6^ total B cells (Fig. 3c), and 10% of all B cells expressing CD80^+^ (Fig. 3i), we calculated that the transferred CD80^+^ cells could have hosted 100 NP-specific memory B cells. Unexpectedly, transfer of memory CD80^+^ B cells from all three tissues generated strong serum IgA and IgG titres, and significant local SI LP IgA plasma cell responses (Fig. 4c,d).

Using the adoptive transfer model with B1-8^high^/GFP^+^ NP-specific B cells, we could also confirm that only p.o. priming immunizations resulted in mucosal memory B cells that could home to the gut SI LP on re-exposure to antigen. Again, we found that p.o. primed mice could be boosted, either p.o. or i.p., to generate a strong SI LP IgA response (Fig. 4e–h; Supplementary Fig. 6a–c). As before, these NP-specific SI LP responses were only observed in p.o. primed mice and not in i.p. primed mice (Fig. 4f). Next, we tested whether the gut homing receptor, α4β7 (Fig. 3k), was required for the SI LP GFP^+^ NP-specific booster response. To this end, we used anti-α4β7 monoclonal antibodies (Mabs) to block gut homing *in vivo*. We found significantly fewer NP-specific GFP^+^ plasma cells in the SI LP in the presence of the blocking antibody after either p.o. or i.p. booster immunizations with NP-CT (Fig. 4i,j; Supplementary Fig. 6d). Thus, expression of the α4β7 receptor on mucosal memory B cells was required for eliciting a plasma cell response in the SI LP.

### Gut memory B cells expand close to or in GCs

**Figure 5:**
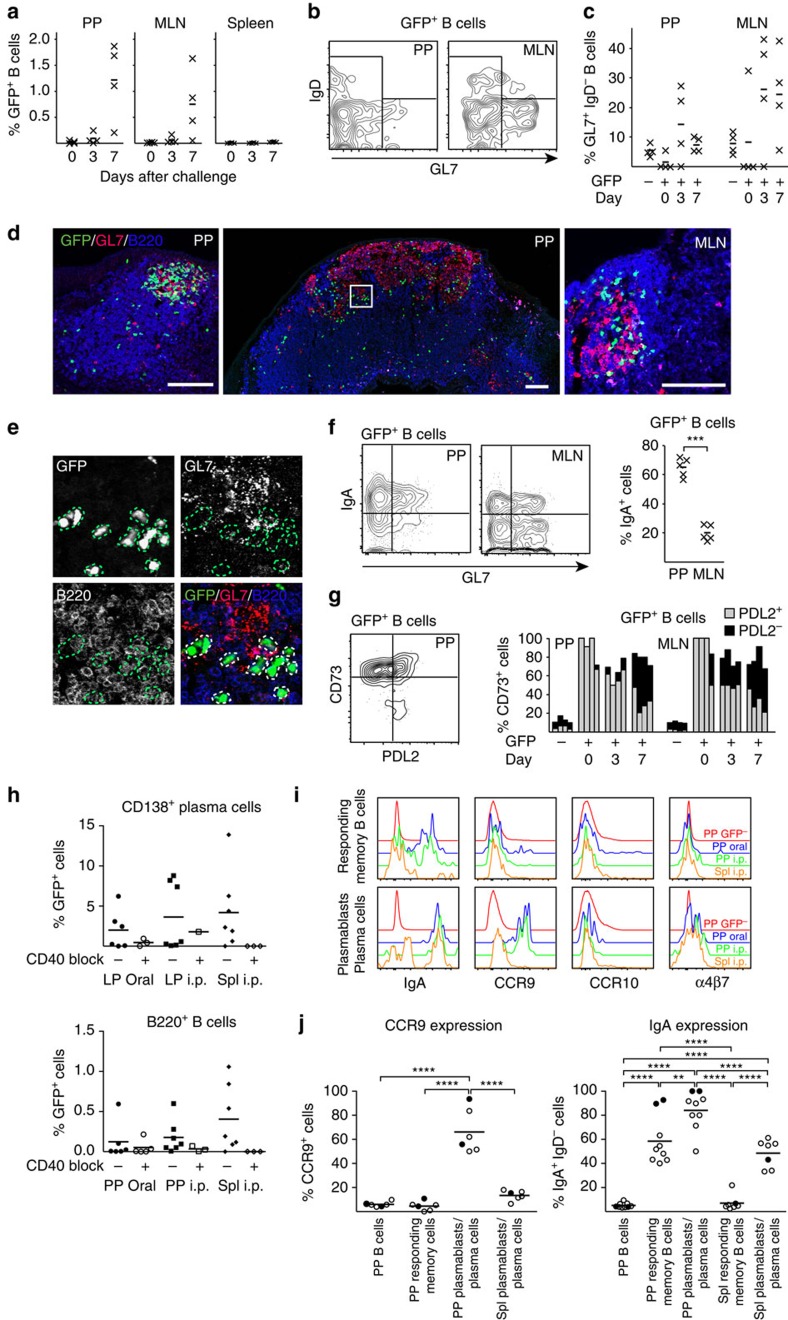
Memory B cells expand in and around pre-existing germinal centres on reactivation, but most cells do not acquire GL7 expression. (**a**) One year after oral priming immunizations, the proportion of NP-specific memory B cells in various tissues was monitored kinetically following a single p.o. challenge immunization with NP-CT. The frequency of NP-specific GFP^+^ cells among total CD19^+^B220^+^CD138^−^ B cells at given time points was determined in individual mice (*x*; protocol as in Fig. 3a). (**b**,**c**) Flow cytometry analysis of GL7 and IgD expression on GFP^+^ memory B cells following the p.o. challenge. (**b**,**c**) The proportion of GFP^+^ cells that were GL7^+^ IgD^−^ was determined using the indicated gates. (**c**) The percentage of GFP^+^ memory B cells that were GL7^+^IgD^−^ was low in the PP, while it was two- to threefold higher in the MLN. (**d**,**e**) Antibody-labelled frozen sections of the PP and MLN were prepared 7 days after the challenge and analysed with confocal microscopy for GL7 (red) and B220 (blue) expression on expanding memory GFP^+^ (green) B cells. (**e**) Close-ups of the PP section marked in the mid panel in **d** with each individual channel shown in black and white and an overlay in colour in which individual GFP^+^ cells are circled to illustrate that most responding GFP^+^ memory B cells express B220, but not the GL7 marker even if located in a germinal centre. (**f**) Flow cytometry analysis on day 7 of IgA expression in responding GFP^+^ memory B cells in the PP and MLN. The percentage of IgA^+^ B cells/all GFP^+^ B cells in the PP or MLN in individual mice is given (right panel). (**g**) Flow cytometry analysis of the expression of CD73 and PD-L2 in responding GFP^+^ CD19^+^B220^+^CD138^−^ memory B cells on different days following a p.o. challenge immunization. Each bar represents the percentage of memory B cells that co-express CD73 and PD-L2 (grey) or only CD73 (black). (**h**) Mice p.o. primed with NP-CT received a p.o. or i.p. challenge immunization 6 months later in the presence or absence of anti-CD40L Mab (clone MR-1), and the proportion of GFP^+^ cells/total B220^+^ B cells or CD138^+^ plasma cells in the lamina propria (LP), spleen (Spl) or PP in individual mice was determined using flow cytometry. The Mann–Whitney non-parametric test showed that GC disruption with anti-CD40L Mab significantly reduced GFP^+^ plasma cell numbers in the SI LP (*P*=0.0193) and responding B-cell numbers in the PP (*P*=0.0053). (**i**) NP-specific GFP^+^ cells in the PP or spleen in p.o. or i.p. challenged mice were divided into responding memory B220^+^ B cells and GFP^high^ B220^−^ plasmablasts/plasma cells (shown in Supplementary Fig. 6e,f), and the expression of IgA, CCR9, CCR10 and integrin α4β7 was determined by flow cytometry. As a control, GFP^−^ B cells from the PP are included in each panel. (**j**) The proportion of mice that express CCR9 or IgA of the indicated GFP^+^ cell populations after an i.p. (open symbols) or p.o. (closed) challenge immunization. Significance was calculated with the Mann–Whitney test (**f**) and analysis of variance followed by Tukey's multiple comparison correction (**j**) and *P* values are given (**0.005≤*P*<0.01, ***0.001≤*P*<0.005, ****P<0.001). Data from four to five mice in each group in one representative experiment out of three giving similar results are shown.

GFP-expressing NP-specific memory B cells increased markedly on an oral NP-CT challenge immunization (Fig. 5a). We observed that already after 3 days were NP-specific GFP^+^ B-cell frequencies significantly increased in the PP and MLN with a continued expansion over the first week (Fig. 5a). Flow cytometry analysis demonstrated that a minority of the GFP^+^ B220^+^CD19^+^CD138^−^ B cells expressed GL7 in either the PP (10–20%) or MLN (25–40%) (Fig. 5b,c; Supplementary Fig. 6a). Nevertheless, most NP-specific memory B220^+^ B cells expanded close to as well as within GL7^+^ GC areas, but without expressing the GL7 marker (Fig. 5d,e). Importantly, 1 week after challenge, ∼70% of all responding GFP^+^ memory B cells in the PP had an IgA^+^IgM^−^IgD^−^ phenotype, whereas in the MLN only 20% were IgA^+^ (80% were IgM^+^ or IgG^+^; Fig. 5f). Moreover, while CD73 and PD-L2 were co-expressed on essentially all resting NP-specific GFP^+^ memory CD19^+^B220^+^CD138^−^ B cells, the PD-L2 marker was downregulated on a majority of responding memory B cells after an oral challenge immunization (Fig. 5g; Supplementary Fig. 6a). Thus, CD73 appeared to be the most reliable marker for identifying resting and activated mucosal memory B cells.

To address whether GC formation was needed for mucosal memory B-cell responses, we boosted p.o. primed mice after 6 months in the presence or absence of a GC-disrupting treatment with an anti-CD40L Mab *in vivo*. We found significantly reduced levels of NP-specific B cells in the PP and of plasma cells in the SI LP following either p.o. or i.p. boosting in anti-CD40L-treated mice (Fig. 5h; Supplementary Fig. 7b–d). Hence, mucosal memory B-cell responses were dependent on GC formation. Both spleen and PP memory B cells expressed the α4β7 homing receptor (Fig. 3k), which raised the question of whether any additional gut homing receptors were acquired in the PP. We found that NP-specific IgA plasmablasts/plasma cells in the PP, but not in the spleen, expressed CCR9 following either i.p. or p.o. boosting, indicating that mucosal memory B cells require first α4β7 and then CCR9 to successfully home to the SI LP (Fig. 5i,j; Supplementary Fig. 7e,f). By contrast, memory B cells residing in the spleen, although expressing α4β7, do not acquire CCR9 and, therefore, appear not to contribute to an IgA plasma cell response in the SI LP.

### Additional clonal selection on antigen reactivation

**Figure 6:**
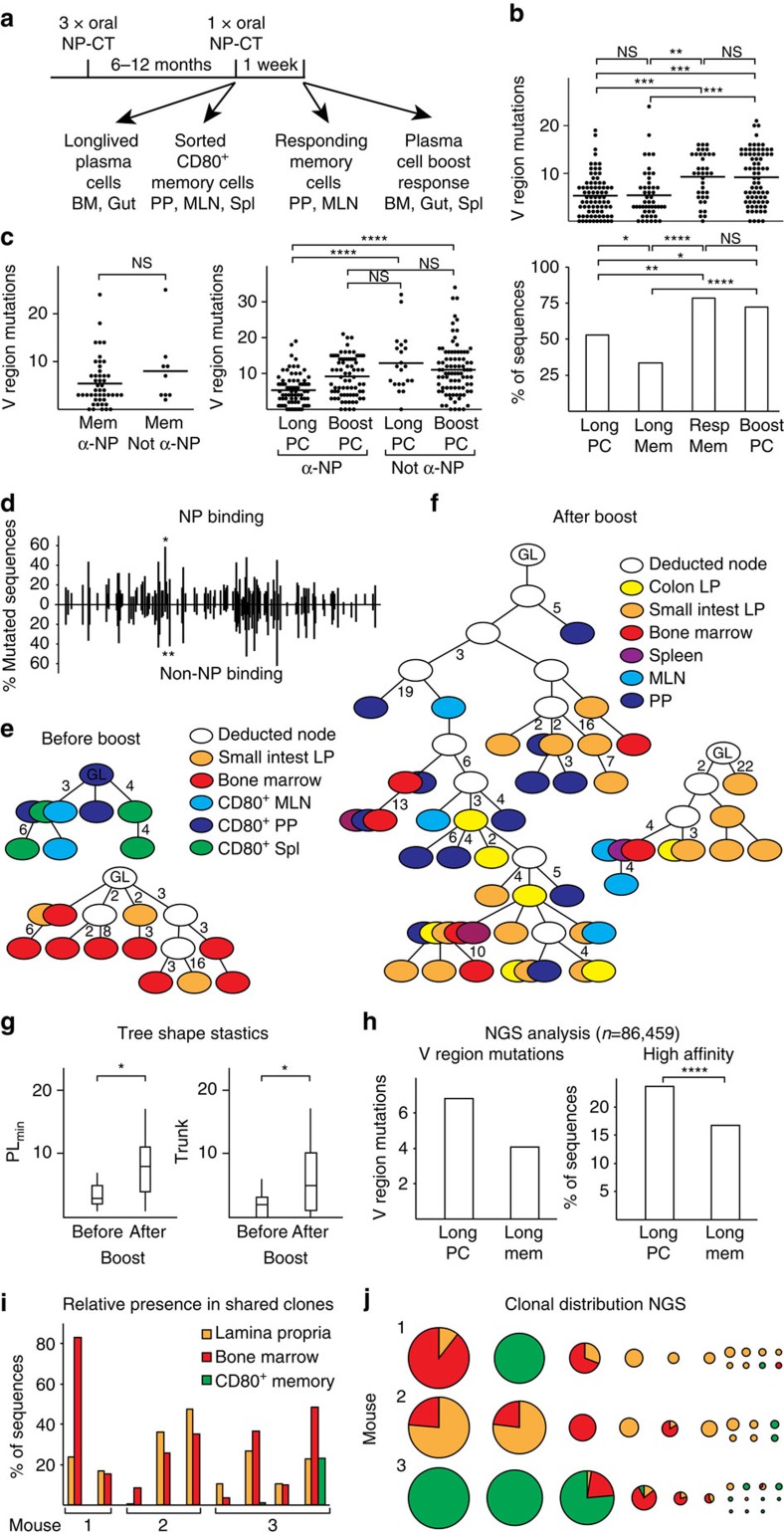
NP-binding IgA V_H_186.2 gene sequence analysis indicates poor relatedness between gut memory B cells and long-lived plasma cells. (**a**) Experimental design. Traditional Sanger sequencing analysis of NP-binding IgA V_H_186.2 genes for mutational load was performed in sorted cells 1 year following three oral priming immunizations. We compared IgA V_H_186.2 gene sequences in long-lived plasma cells (Long PC) with those of CD80-expressing memory B cells (Long Mem) and IgA V_H_186.2 gene sequences in responding memory B cells (Resp Mem) with those of SI LP plasma cells (Boost PC) 10 days after boosting (Figs 1d,e and 2e,f; Supplementary Fig. 7a,b). (**b**) The number of mutations (individual and mean) in the V_H_186.2 gene region (upper panel) and the proportion of gene sequences with high-affinity mutations (lower panel). (**c**) The number of mutations in the V_H_186.2 region of NP-binding sequences was compared with those found in non-NP-binding V_H_186.2 sequences. Sorted CD80^+^ memory B cells (left panel) and orally primed IgA plasma cells before (Long) and after booster (Boost) (right panel) are included. *P* values are given as in **b**. (**d**) Distribution of mutations in NP-binding (above the line) and non-NP-binding V_H_186.2V regions (below the line) are shown. Affinity-enhancing CDR1 mutations at position 98 and the counter-selected mutation in 102 are marked with one or two stars, respectively. (**e**,**f**) Examples of clonal trees generated using the IgTree program^48^ from individual mice before (**e**; *n*=3) and after (**f**; *n*=6) an oral booster with NP-CT are shown. (**g**) Tree shape statistics^33^ were analysed and significant differences were observed in PL_min_ (minimum tree path length) and trunk length in trees constructed from NP-binding V_H_186.2 gene sequences from mice before and after a booster immunization. Pooled data from three independent experiments with three to six mice per group. (**h**) Targeted NGS sequencing of NP-specific IgA V_H_186 genes expressed in sorted long-lived plasma cells in the SI LP and BM or sorted memory CD80^+^ B cells from individual mice (*n*=3). Figures represent mean mutation rates and frequencies of high-affinity mutations analysed in 86,000 NP-binding sequences. For **b**,**c**,**g** and **h**, the Mann–Whitney test *P* values are given (not significant (NS) *P*>0.05, *0.01≤*P*<0.05, **0.005≤*P*<0.01, ***0.001≤*P*<0.005, ****P≤0.001). (**i**,**j**) NGS sequences from IgA long-lived plasma cells and CD80^+^ memory B cells from three individual mice were divided into clones (described in Supplementary Fig. 9). (**i**) Relative proportion of sequences generated from each mouse that were present in shared clones (for example, two clones from mouse 1 contained sequences from both the LP and BM, and in the first clone 83% of all sequences from the BM and 24% from the LP were present). (**j**) The distribution of sequences between clones (clones with only one sequence excluded). The actual number of sequences allocated to a clone is indicated by the size of the pie chart, and the distribution of origin of the sequences within the pie chart with colours.

Finally, we compared the mutational load and affinity maturation in NP-binding IgA V_H_186.2 V gene sequences following oral priming immunizations. We compared (1) sorted CD80^+^ memory B cells from the PP and BM (Supplementary Fig. 8a), with (2) long-lived SI LP plasma cells (Fig. 1d,e), or (3) responding memory B cells from the PP and MLN (Supplementary Fig. 8b) with (4) plasma cells from the SI LP following an oral NP-CT booster immunization (Fig. 2e,f). Using traditional Sanger sequencing, we observed that while NP-binding memory B cells and long-lived plasma cells had similar numbers of V region mutations, significantly more of the latter carried high-affinity CDR1 mutations. This suggested that they had undergone similar divisions in the GC, but that long-lived plasma cells were subjected to a stronger selection process or, in fact, were generated from uniquely different GC reactions (Fig. 6b). In line with the latter interpretation, only 3 out of 48 memory B-cell IgA V_H_186.2V gene sequences demonstrated clonal relationships with those analysed from SI LP long-lived plasma cells in the same mouse (Fig. 6e). Following an oral NP-CT booster immunization, the responding memory B cells and SI LP plasma cells had increased mutation frequencies in the NP-binding IgA V_H_186.2V gene and were of higher affinity than the resting memory B cells and long-lived plasma cells (Fig. 6b). It, thus, appeared that reactivation of memory B cells resulted in an efficient selection and affinity maturation process. Moreover, long-lived NP-specific plasma cells had fewer mutations than non-NP-binding plasma cells with rearranged V_H_186.2V genes, but a challenge immunization increased the former's mutational load to the same level as the latter (Fig. 6c). As expected, no significant change in mutation frequencies in non-NP-binding cells were detected following a booster immunization (Supplementary Fig. 8c). The distribution of mutations in non-NP and NP-binding V_H_186.2V regions did not reveal any selected NP hotspots except for the CDR1 affinity-enhancing mutation at position 98 (Fig. 6d). Interestingly, a nearby position, 102, was more commonly mutated in non-NP- than NP-binding sequences (42% versus 27.5% of the sequences), and, based on clonal tree analysis, was never mutated before the 98 position in NP-binding sequences.

Clonal trees analysis from individual mice further supported the observation that NP-specific memory B cells and long-lived plasma cells were poorly related (Fig. 6e; Supplementary Fig. 7d). By contrast, responding NP-specific IgA memory B cells in the PP and MLN exhibited strong clonal relationships with IgA plasma cells in the LP or BM (Fig. 6f; Supplementary Fig. 8e). Only 1 out of 10 trees generated from orally primed, but not boosted, mice exhibited related gene sequences between plasma cells and memory B cells, whereas 8 out of 13 trees from primed and boosted mice shared gene sequences between IgA plasma cells and responding memory B cells from the PP or MLN. Bootstrapping analysis of the data confirmed that the poor relatedness and the experimentally observed numbers of mixed clones were significantly different from that obtained under random sequence assignment (*P*<0.05, *z*-test, 10,000 simulation runs per mouse group). Furthermore, two parameters describing tree shape statistics were found significantly different, PL_min_ (minimum tree path length) and trunk length, between trees generated before and after booster immunizations, which further supported the view that memory B cells undergo additional selection or maturational processes in response to oral booster immunizations (Fig. 6g; Supplementary Fig. 8f)^35^.

Next-generation sequencing (NGS) of antibody genes in long-lived plasma cells and memory B cells confirmed these observations (Fig. 6h–j; Supplementary Fig. 9). In over 86,000 NP-specific gene sequences, more mutations and higher levels of affinity maturation were observed in IgA-expressing long-lived plasma cells than in IgA memory B cells (Fig. 6h). When sequences were ordered into clones, only one out of three mice showed any clonal overlap between memory B cells and long-lived plasma cells, and even in this mouse, less than one in four memory sequences were present in clones that also hosted long-lived plasma cell sequences (Fig. 6i,j). In contrast, large clones with overlap between LP and BM plasma cells were found in all mice. Taken together, the sequence and lineage tree analysis data suggest that memory B cells and long-lived plasma cells were poorly related, and that the former leave GC reactions at an earlier time point than the plasma cells. Furthermore, boosted memory B cells and subsequent IgA long-lived plasma cells in the LP and BM are strongly clonally related, arguing for a highly effective selection and maturation of memory B cells in GC. This mechanism is likely to secure the production of only high-quality IgA antibodies in the gut LP following re-exposure to the antigen.

## Discussion

Unexpectedly, we found that oral priming immunizations induced long-lived plasma cells and memory B cells that showed poor clonal relatedness, indicating that they emanated from a unique or kinetically separate induction process in GALT. This notion finds support from recent studies by Tas *et al*. and Weisel *et al*., who documented the gradual disappearance of clones and a shift from the formation of memory to plasma cells during the GC reaction^36^,^37^. A majority of memory B cells in the PP carried IgA and following a booster immunization these cells expanded close to or in GC, which was critical for selecting and affinity-maturing the repertoire of gut IgA plasma cells in the small intestine. While in very low frequencies, ∼0.01–0.02‰ of all B cells, memory B cells exhibited exceptional expansion on reactivation and underwent effective selection and maturation. Irrespective of the source, PP, MLN or spleen, CD80^+^ memory B cells with gut homing properties gave rise to significant SI LP IgA AFCs. Although the GC reaction was found critical, only few responding memory B cells expressed GL7. Still, a detailed analysis of tissue sections confirmed their presence in high densities in GC, at least in the PP. Therefore, we believe that GL7 expression is not a reliable marker for GC B cells in the PP. Studies are ongoing to further investigate this observation, analysing light and dark zone B cells as well as the correlation between GL7 expression and the differentiation into plasma cells, as has been proposed^38^,^39^.

A unique property of the PP as a secondary lymphoid tissue is the constant presence of GC^15^. Therefore, GCs in the PP could be viewed as sites for persistent GC functions, reminiscent of the persistent GC-like structures observed after systemic immunizations with sheep red blood cells^17^. However, in contrast to systemic sheep red blood cell immunizations, or the MLN and spleen after oral immunizations in our study, where IgM^+^ memory B cells dominated, the PP hosted predominantly IgA^+^ memory B cells. The PP IgA^+^ memory B cells responded to recall antigen, expanded in GC, acquired additional mutations and differentiated into IgA plasma cells that subsequently were found in the SI LP. Notably, this scenario contrasts with that described by Dogan *et al*. who found that it was mostly IgM^+^ memory B cells that expanded and differentiated in the persistent GC-like structures, while switched memory B cells rapidly matured into plasma cells^17^. However, the dependence on GC for gut IgA memory responses was further supported by experiments performed in anti-CD40L Mab-treated mice, which exhibit disrupted GC functions. These experiments demonstrated markedly reduced boosting effects of mucosal memory B cells in the PP and a subsequent lack of antigen-specific IgA plasma cells in the SI LP. Therefore, we propose that the GC environment in PP is quite unique in that it allows expansion, selection and differentiation of isotype-switched memory B cells, while at least two other studies have ascribed this property to IgM^+^ memory B cells^17^,^18^. More recently, Zuccarin-Catania *et al*. provided evidence that it may not be the IgM isotype that explains why memory B cells can re-engage in GC, but rather the expression of CD80 and PD-L2 (ref. 19). This seems logical, but our observation that CD80^+^PD-L2^+^ IgA^+^ memory B cells expanded in GC in the PP conflicts with the finding of Zuccarin-Catania *et al*. who reported that primarily the double-negative memory B cells populated secondary GCs. We found that PD-L2 was downmodulated on IgA^+^ memory B cells during booster responses, but CD73 was retained. Hence, the expression of CD73^+^ on gut B cells appears to be a good indicator of GC involvement. Notably, we used CD80 to enrich for memory B cells before transfer into μMT mice, and we found strong serum and SI LP IgA as well as serum IgG responses. This was observed despite the fact that a majority of the transferred memory B cells were unswitched IgM^+^ memory B cells. We did not investigate GC reactions in recipient μMT mice, but it is likely that they reflected both early GC-independent and secondary GC-dependent responses. Nevertheless, although we did not directly address how CD80, CD73 and PD-L2 expression influenced the antigen-specific memory B-cell response, our data complement and support that of Zuccarin-Catania *et al*. in showing that isotype-switched IgA^+^ memory B cells can engage in GC reactions^19^. This is further supported by recent studies by McHeyzer-Williams *et al*.^40^ who demonstrated, at the single-cell level, reinitated BCR diversification in switched memory B cells in secondary GC.

We found that NP-specific memory B cells were rare at 1 year after oral immunization. However, these rare memory B cells rapidly expanded and made up on average 15% of all plasma cells in the gut LP within a week after an oral challenge immunization. We calculated that as few as 1 in 100,000 B cells could respond to NP in orally NP-CT primed mice and, hence, as we transferred 75,000–100,000 sorted CD80^+^ B cells each mouse would have received between 100 and 200 NP-specific and three- to fivefold more CT-specific memory B cells. We observed that 80–100% of the μMT mice responded to CT and ∼40% responded to NP after an oral challenge immunization. Importantly, irrespective of whether the memory B cells were isolated from the PP, MLN or spleen, the progeny gave rise to a strong IgA plasma cell response in the SI LP following oral immunization. This argues that memory B cells in mucosal as well as systemic locations carried gut homing properties following oral priming immunizations. By contrast, this was not the case after parenteral priming immunizations. We found that GFP^+^ memory B cells in the spleen exhibited enhanced expression of α4β7 only after oral priming immunizations. In analogy to memory B cells after intestinal rotavirus infection, mucosal homing receptors α4β7 were a pre-requisite for transferring gut IgA plasma cell responses into recipient mice^10^. Rotavirus-specific memory B cells that did not express α4β7 failed to transfer SI LP IgA immunity, but they effectively transferred a systemic IgG response^10^. The present study further complements this picture, by confirming that anti-α4β7 Mab blockade strongly reduced homing to the SI LP of NP-specific IgA cells following p.o. as well as i.p. booster immunizations.

Whether memory B cells are sessile or migrating is of fundamental importance for explaining the observation that memory B cells from the spleen can populate SI LP as IgA plasma cells after expansion. Our data would lead us to hypothesize that both mucosal and systemic memory B cells are, indeed, re-circulating cells following oral priming. The progressive accumulation of somatic mutations in IgA V region genes, shown by Lindner *et al*., and the present finding that B cells with a memory CD73^+^ phenotype increase with age in the PP, MLN and spleen support the notion that memory B cells are re-circulating cells^6^,^7^. Of note, a large number of IgA-expressing cells with a memory phenotype circulate in human blood^41^,^42^. Although memory B cells expressing the gut homing receptor α4β7 reside in the spleen following oral immunizations, we propose that only when entering the PP (and possibly the MLN) can such memory B cells upregulate expression of CCR9 on reactivation and in this way home to the gut LP and give rise to IgA plasma cells. Thus, both α4β7 and CCR9 expression are required for memory B-cell responses to locate to the SI LP. To explain how our transfer of predominantly IgM^+^CD80^+^ memory B cells from spleen could result in IgA plasma cell responses in gut LP, we would have to suggest that these cells migrated to the PP where they underwent IgA class switch recombination and upregulated CCR9, on reactivation. Moreover, IgG (BM) and IgA (SI) long-lived plasma cells, while being clonally related, exhibited distinct tissue distribution following oral priming immunizations. This could also be explained on the basis of acquisition of differential homing receptors, which may occur in the PP or MLN after leaving the GC^43^.

Future investigations into gut IgA memory B-cell responses after oral immunizations are necessary and critically important for mucosal vaccine design and development. The observation that gut memory B cells and long-lived plasma cells were found poorly related is intriguing. This finding was obtained not only with traditional Sanger sequencing, but, more importantly, was confirmed by NGS of >86,000 NP-specific IgA sequences in three individual mice. Only in one mouse did we find evidence of clonal overlap between memory B cells and long-lived plasma cells, and this was only found with 25% of the memory sequences analysed in this mouse. Moreover, the significance of that only 3 out of 48 memory B-cell clones of the IgA V_H_186.2V gene showed an overlap with sequences found in long-lived plasma cells was also subjected to bootstrapping simulations and the results were found highly significant. Therefore, we propose a model in which plasticity of the GALT allows for the generation of memory B cells concomitant with the generation of long-lived plasma cells, but involving temporarily separate processes in the GC or even anatomically separate GC, leading to lower-affinity maturation in memory B cells. This allows for a broader, less mutated, repertoire of both IgM^+^ and IgA^+^ mucosal memory B cells following oral immunization than that ultimately used for host protection against, for example, an intruding pathogen. However, on re-exposure to antigen, gut memory B cell clones have migrated to multiple sites in the GALT and the response can be synchronized and clones strongly selected, leading to pronounced clonal relatedness in the GALT and SI LP with only high-affinity and strongly mutated clones being represented. It can be envisioned that a broad memory B-cell repertoire is functionally important and could, for example, explain cross-protection against related pathogens, as seen between cholera and enterotoxic *Escherichia coli* infections^44^.

## Methods

### Mice and immunizations

All experiments were carried out under the ethical permit 1/14 and initiated when female mice reached the age of 6–10 weeks. Apart from C57BL/6 mice (Taconics, Bomholt, Denmark), we used for NP-specific B-cell experiments F1 mice generated through crossing C57BL/6 mice with homozygous B1-8^high^GFP mice^45^ (a kind gift from M Nussenzweig, Rockefeller University, New York, NY). These mice and μMT mice, both on a C57BL/6 background, were bred and housed under specific pathogen-free conditions at the animal facility Experimental Biomedicine (EBM) at the University of Gothenburg. NP-specific GFP^+^ splenic λ-expressing B cells were prepared through depletion of non-B cells and κ-expressing cells using an EasySep Mouse B cell isolation kit (Stem Cell Technologies, Manchester, UK) supplemented with 2 μg anti-mouse κ-chain biotinylated antibody (BD Biosciences, San Jose, CA). For oral immunizations, a dose of 20 μg of NP-CT was used. In brief, CT was dialysed in distilled water for 2 days before mixing it with an equal volume of 0.1 m NaHCO_3_ and 20 equiv. NP-OSu (Biosearch Technologies, Novato, CA) per mole CT. The mixture was incubated for 12 h at 4 °C and transferred into a Slide-A-Lyzer dialysis cassette and dialysed against 0.05 M NaHCO_3_, followed by water before the protein concentration was determined using a BCA assay (Thermo Fisher Scientific, Rockford, IL)^29^. Three oral priming immunizations were performed 10 days apart followed by a single booster dose 1 year after the priming. I.p. immunizations were performed in wild-type mice with 5 μg NP-CGG plus 5 μg of CTA1-DD adjuvant and in the adoptive B1-8^high^GFP B-cell transfer model, we used 1–2 μg NP-CT. For blocking gut homing, we used the antibody clone DATK32 specific for the integrin α4β7 (Bio X Cell, West Lebanon, NH); 250 μg of the antibody was injected i.p. 3 days before, on the day of and 3 days after booster immunization. The anti-CD40L-binding clone MR-1 was used for CD40 blockade using the same protocol (Bio X Cell).

### Enzyme-linked immunospot

Ninety-six-well plates (Merck Millipore, Billerica, MA) were coated with 5 μg ml^−1^ rat anti-mouse IgA antibodies (BD), 5 μg ml^−1^ goat anti-mouse IgG antibodies (Southern Biotech, Birmingham, AL), 3 nmol ml^−1^ GM1-ganglioside followed by 3 μg ml^−1^ CT or 10 μg ml^−1^ NP-bovine serum albumin (BSA; Biosearch Technologies, Novato, CA) in PBS at 4 °C overnight. The plates were washed in PBS, blocked with 0.1% BSA in PBS and 10^4^–10^6^ purified cells from the indicated organs were added in duplicates and diluted 1:3 in four serial dilutions and incubated at 37 °C for 3 h. AP-conjugated goat anti-mouse IgA and IgG antibodies (Southern Biotech) were added and incubated overnight at 4 °C. AFCs were visualized using BCIP substrate (Sigma-Aldrich, St Louis, MO) and analysed using an Immunospot ELISPOT system (Cellular Technology Ltd, Shaker Heights, OH).

### Enzyme-linked immunosorbent assay

Ninety-six-well plates (Thermo Fisher Scientific, Rockford, IL) were coated with 100 μl per well of 10 μg ml^−1^ NP-BSA or 0.5 nM ml^−1^ GM1 followed by 0.5 μg ml^−1^ CT overnight at 4 °C. The plates were treated as for enzyme-linked immunospot and serum samples were diluted 1:100 or gut lavages were diluted 1:10 and two- or threefold serial dilution series were performed. Samples were incubated overnight at 4 °C, followed by isotype-specific AP-conjugated rat anti-mouse antibodies (Southern Biotech) at a dilution of 1:1,000 in PBS/0.1% BSA. The plates were developed using NPP substrate (Sigma-Aldrich) and analysed at 405 nm using a Titertek Multiscan (MTX Labsystems, Vienna, VA) spectrophotometer.

### Immunohistochemistry

Mice were killed, and the intestine, PP and MLN were embedded in TissueTek OCT compound and snap-frozen in liquid nitrogen. Tissues containing GFP-expressing cells were fixed in 4% paraformaldehyde and 10% sucrose for 1 h, followed by 30% sucrose/PBS overnight before freezing. Frozen sections (7 μm) were fixed in 100% acetone and blocked with 5% normal horse serum in PBS for 15 min. Antibodies used to stain sections were GL7-eFlour 660 (1:200, 50-5902, eBiosciences, San Diego, CA) and B220-biotin (1:500, 553086, BD Bioscience), followed by Streptavidin-Alexa Flour 594 (1:150, S11227, Life Technologies) or CD138 PE (1:100, 553714, BD Bioscience) and IgA-biotin (1:100, 556978, BD Bioscience), followed by Streptavidin-Alexa Flour 647 (1:150, S32357, Life Technologies). Microscopy was performed at the Centre for Cellular Imaging using the confocal Zeiss LSM 700 inv system and LSM software (Carl Zeiss, Oberkochen, Germany).

### Flow cytometry and cell sorting

Lymphocytes were isolated as described and stained with anti-mouse IgD PerCP-Cy5.5 (1:400, 405710), LPAM (integrin α4β7) PE (1:100, 120605; (Biolegend, San Diego, CA), B220 V500 (1:400, 561227), CD19 APC-H7 (1:200, 560143), CD80 PE (1:500, 553769), CD273 APC (1:200, 560086), CD138 PE (1:200, 553714), IgM PE-Cy7 (1:200, 552867; BD Biosciences), CCR9 PE-Cy7 (1:100, 25-1991), CD73 PE-Cy7 (1:50, 25-0731), IgA PE (1:50, 12-4204), GL7 eFluor 450 (1:100, 48-5902), CD38 Alexa700 (1:800, 56-0381), CD21/35 Pacific Blue (1:800, 57-0212; eBiosciences) or CCR10 APC (1:100, FAB2815A; R and D systems. Minneapolis, MN) and were analysed using LSR II (BD Biosciences) or Navios (Beckman Coulter, Brea, CA) flow cytometers. For sorting, cells were labelled with anti-mouse CD138 PE (1:200, 553714), CD19 PE-Cy7 (1:200, 552854), CD80 APC (1:200, 560016) and GL7 FITC (1:100, 553666) before sorting using a FACSAria (BD Biosciences). Cells were sorted into tubes that had been coated with 2% BSA/PBS overnight, and pelleted by centrifugation at 600 *g* before being resuspended in PBS and injected into recipient mice or used for gene sequence analysis.

### Cloning and sequencing

FACS-sorted CD80^+^CD138^−^ B cells, single-cell suspensions from the spleen, MLN, PP or BM, or 1 cm pieces of the colon or small intestine were prepared and submerged in 350 μl RLT buffer (Qiagen, Hilden, Germany). The tissue was disrupted and homogenized and RNA extracted using RNeasy mini or micro kits (Qiagen). cDNA synthesis was performed using an IgA- (5′-TGACATTGGTGGGTTTAC-3′) or IgM- (5′-GAGGAAGAGGACGATGAA-3′) specific primer and Superscript III RT polymerase (Life Technologies, Carlsbad, CA) at 50 °C for 1 h. PCR primers were: IgA NP PCR, IgA down2 (5′-TTCCTCGAGGGATGGCAGGAAGGAGGAC-3′), NP leader up (5′-TCTAGAATTCGGGATGGAGCTGTATCATGCTC-3′). All PCRs were carried out with an annealing temperature of 64 °C using standard PCR protocols with 30 cycles using the high-fidelity Phusion enzyme (Thermo Fisher Scientific). The PCR reactions were cloned into ZeroBlunt TOPO vectors (Invitrogen), transformed into bacteria and single colonies were identified by colony hybridization using a probe for NP-specific antibody transcripts (5′-CCGTAGTAATATCTTGCACA-3′) end-labelled with P^32^ after transfer to a Hybond membrane (GE Healthcare, Piscataway, NJ). Hybridization to colonies was detected on a ^32^P-sensitive phosphorus screen and analysed using the PharosFX plus system (Bio-Rad, Hercules, CA). Plasmid minipreps were prepared using GeneElute kits (Sigma) from overnight cultures.

When traditional Sanger sequencing was used (Eurofins MWG Operon, Ebersberg, Germany), clones were analysed using the Staden package^46^. Clones were classified as NP binding if their CDR3 region was between 9 and 11 amino acids long, they had a tyrosine at position 99, and there were at least two more tyrosine residues in the following three amino acids. When NGS of NP-binding gene sequences was undertaken, the Ion Torrent platform was used. In brief, cells were prepared and IgA cDNA was made exactly as described above, and each sample was subjected to two parallel 25-cycle PCR reactions using primers as above. PCR products were pooled and purified using a MinElute PCR purification kit (Qiagen), and 2 μl eluted DNA was used in nested PCR reactions with FRW1 and IgA primers (5′-AAGCTGTCCTGCAAGGCTTC-3′ and 5′-CTTGACAGAGCTCGTGGGAG-3′) with barcodes, A and trP1 sequences attached to primers as described in Ion Amplicon Library Preparation (Fusion Method; Publication number 4468326, revision C available from ThermoFisher) using 25 cycles for PCR amplification. After purification of PCR product using the MinElute PCR purification kit (Qiagen) and quantification using a Qubit 2.0 fluorometer (ThermoFisher), four upstream and four downstream reactions with unique barcodes were loaded at 40 pM concentration onto Ion 314 v2 Chips using an Ion Chef and were sequenced, using the 400 bp IonPGM HiQ Sequencing protocol according to the instructions. Sequences were aligned to V, D and J segments using IMGT/HighV-QUEST^47^ and further processed as described in Supplementary Fig. 9. Noteworthy, the NGS technique resulted in a lower frequency of mutations and gene sequences carrying the high-affinity mutation than the Sanger technique (b), probably as a consequence of that many highly mutated sequences were not amplified in the nested PCR reaction due to mutations interfering with PCR primer binding in FRW1.

### Statistical analysis and bootstrapping simulation

Values are given ±s.d. and the number of experiments and group sizes are given in the figure legends. Analyses of significance were done with the Mann–Whitney test using the Prism software (GraphPad). Error bars represent values of s.d. All reported *P* values are two-sided and values of <0.05 were considered to indicate statistical significance (not significant (NS) *P*>0.05, *0.01≤*P*<0.05, **0.005≤*P*<0.01, ***0.001≤*P*<0.005, ****P≤0.001).

Each run of the bootstrapping simulations took the same number of cells in each group of mice as in the experiments, and divided them randomly into clones (with the same total number of clones as in each group in the experiments), and asked whether we get similar numbers of mixed clones to those obtained in the experiments. The simulations were run 10,000 times, and the *z*-test was used to compare their results with the experimental data. Values of <0.05 were considered to indicate statistical significance.

### Data availability

NGS data have been deposited at the Gene Expression Omnibus database under accession code GSE84698.

## Additional information

**How to cite this article:** Bemark, M. *et al*. Limited clonal relatedness between gut IgA plasma cells and memory B cells after oral immunization. *Nat. Commun.* 7:12698 doi: 10.1038/ncomms12698 (2016).

## Supplementary Material

Supplementary Information Supplementary Figures 1-9

## References

[b1] Harris A. M. . Antigen-specific memory B-cell responses to *Vibrio cholerae* O1 infection in Bangladesh. Infect. Immun. 77, 3850–3856 (2009). 19528207 10.1128/IAI.00369-09 PMC2738048

[b2] Narváez C. F. . Human rotavirus-specific IgM Memory B cells have differential cloning efficiencies and switch capacities and play a role in antiviral immunity *in vivo*. J. Virol. 86, 10829–10840 (2012). 22855480 10.1128/JVI.01466-12 PMC3457288

[b3] Quiding M. . Intestinal immune responses in humans. Oral cholera vaccination induces strong intestinal antibody responses and interferon-gamma production and evokes local immunological memory. J. Clin. Invest. 88, 143–148 (1991). 1905327 10.1172/JCI115270 PMC296014

[b4] Pasetti M. F., Simon J. K., Sztein M. B. & Levine M. M. Immunology of gut mucosal vaccines. Immunol. Rev. 239, 125–148 (2011). 21198669 10.1111/j.1600-065X.2010.00970.x PMC3298192

[b5] Hapfelmeier S. . Reversible microbial colonization of germ-free mice reveals the dynamics of IgA immune responses. Science 328, 1705–1709 (2010). 20576892 10.1126/science.1188454 PMC3923373

[b6] Lindner C. . Age, microbiota and T cells shape diverse individual IgA repertoires in the intestine. J. Exp. Med. 209, 365–377 (2012). 22249449 10.1084/jem.20111980 PMC3280880

[b7] Lindner C. . Diversification of memory B cells drives the continuous adaptation of secretory antibodies to gut microbiota. Nat. Immunol. 16, 880–888 (2015). 26147688 10.1038/ni.3213

[b8] Lycke N. & Holmgren J. Intestinal mucosal memory and presence of memory cells in lamina propria and Peyer's patches in mice 2 years after oral immunization with cholera toxin. Scand. J. Immunol. 23, 611–616 (1986). 3704560 10.1111/j.1365-3083.1986.tb01995.x

[b9] Lycke N., Hellström U. & Holmgren J. Circulating cholera antitoxin memory cells in the blood one year after oral cholera vaccination in humans. Scand. J. Immunol. 26, 207–211 (1987). 3498210 10.1111/j.1365-3083.1987.tb02253.x

[b10] Williams M. B. . The memory B cell subset responsible for the secretory IgA response and protective humoral immunity to rotavirus expresses the intestinal homing receptor, alpha4beta7. J. Immunol. 161, 4227–4235 (1998). 9780197

[b11] Mesin L., Di Niro R., Thompson K. M., Lundin K. E. A. & Sollid L. M. Long-lived plasma cells from human small intestine biopsies secrete immunoglobulins for many weeks *in vitro*. J. Immunol. 187, 2867–2874 (2011). 21841131 10.4049/jimmunol.1003181

[b12] Lycke N. & Bemark M. Mucosal adjuvants and long-term memory development with special focus on CTA1-DD and other ADP-ribosylating toxins. Mucosal Immunol. 3, 556–566 (2010). 20844480 10.1038/mi.2010.54

[b13] Blutt S. E., Miller A. D., Salmon S. L., Metzger D. W. & Conner M. E. IgA is important for clearance and critical for protection from rotavirus infection. Mucosal Immunol. 5, 712–719 (2012). 22739233 10.1038/mi.2012.51 PMC3461240

[b14] Lycke N. Recent progress in mucosal vaccine development: potential and limitations. Nat. Rev. Immunol. 12, 592–605 (2012). 22828912 10.1038/nri3251

[b15] Bemark M., Boysen P. & Lycke N. Y. Induction of gut IgA production through T cell-dependent and T cell-independent pathways. Ann. NY Acad. Sci. 1247, 97–116 (2012). 22260403 10.1111/j.1749-6632.2011.06378.x

[b16] Tarlinton D. & Good-Jacobson K. Diversity among memory B cells: origin, consequences, and utility. Science 341, 1205–1211 (2013). 24031013 10.1126/science.1241146

[b17] Dogan I. . Multiple layers of B cell memory with different effector functions. Nat. Immunol. 10, 1292–1299 (2009). 19855380 10.1038/ni.1814

[b18] Pape K. A., Taylor J. J., Maul R. W., Gearhart P. J. & Jenkins M. K. Different B cell populations mediate early and late memory during an endogenous immune response. Science 331, 1203–1207 (2011). 21310965 10.1126/science.1201730 PMC3993090

[b19] Zuccarino-Catania G. V. . CD80 and PD-L2 define functionally distinct memory B cell subsets that are independent of antibody isotype. Nat. Immunol. 15, 631–637 (2014). 24880458 10.1038/ni.2914 PMC4105703

[b20] Bergqvist P., Stensson A., Lycke N. Y. & Bemark M. T cell-independent IgA class switch recombination is restricted to the GALT and occurs prior to manifest germinal center formation. J. Immunol. 184, 3545–3553 (2010). 20207993 10.4049/jimmunol.0901895

[b21] Bos N. A. . Serum immunoglobulin levels and naturally occurring antibodies against carbohydrate antigens in germ-free BALB/c mice fed chemically defined ultrafiltered diet. Eur. J. Immunol. 19, 2335–2339 (1989). 2606142 10.1002/eji.1830191223

[b22] Macpherson A. J. . A primitive T cell-independent mechanism of intestinal mucosal IgA responses to commensal bacteria. Science 288, 2222–2226 (2000). 10864873 10.1126/science.288.5474.2222

[b23] Pabst O. New concepts in the generation and functions of IgA. Nat. Rev. Immunol. 12, 821–832 (2012). 23103985 10.1038/nri3322

[b24] Kawamoto S. . Foxp3(+) T cells regulate immunoglobulin a selection and facilitate diversification of bacterial species responsible for immune homeostasis. Immunity 41, 152–165 (2014). 25017466 10.1016/j.immuni.2014.05.016

[b25] Hirota K. . Plasticity of TH17 cells in Peyer's patches is responsible for the induction of T cell-dependent IgA responses. Nat. Immunol. 14, 372–379 (2013). 23475182 10.1038/ni.2552 PMC3672955

[b26] Lycke N. Y. & Bemark M. The role of Peyer's patches in synchronizing gut IgA responses. Front. Immunol. 3, 329 (2012). 23181060 10.3389/fimmu.2012.00329 PMC3500999

[b27] Lycke N. & Holmgren J. Long-term cholera antitoxin memory in the gut can be triggered to antibody formation associated with protection within hours of an oral challenge immunization. Scand. J. Immunol. 25, 407–412 (1987). 3576134 10.1111/j.1365-3083.1987.tb02207.x

[b28] Czerkinsky C., Svennerholm A. M., Quiding M., Jonsson R. & Holmgren J. Antibody-producing cells in peripheral blood and salivary glands after oral cholera vaccination of humans. Infect. Immun. 59, 996–1001 (1991). 1997444 10.1128/iai.59.3.996-1001.1991 PMC258358

[b29] Gärdby E. . Strong differential regulation of serum and mucosal IgA responses as revealed in CD28-deficient mice using cholera toxin adjuvant. J. Immunol. 170, 55–63 (2003). 12496383 10.4049/jimmunol.170.1.55

[b30] Bergqvist P. . Re-utilization of germinal centers in multiple Peyer's patches results in highly synchronized, oligoclonal, and affinity-matured gut IgA responses. Mucosal Immunol. 6, 122–135 (2013). 22785230 10.1038/mi.2012.56

[b31] Vajdy M., Kosco-Vilbois M. H., Kopf M., Köhler G. & Lycke N. Impaired mucosal immune responses in interleukin 4-targeted mice. J. Exp. Med. 181, 41–53 (1995). 7807021 10.1084/jem.181.1.41

[b32] Mattsson J. . Complement activation and complement receptors on follicular dendritic cells are critical for the function of a targeted adjuvant. J. Immunol. 187, 3641–3652 (2011). 21880985 10.4049/jimmunol.1101107

[b33] Anderson S. M., Tomayko M. M., Ahuja A., Haberman A. M. & Shlomchik M. J. New markers for murine memory B cells that define mutated and unmutated subsets. J. Exp. Med. 204, 2103–2114 (2007). 17698588 10.1084/jem.20062571 PMC2118690

[b34] Tomayko M. M., Steinel N. C., Anderson S. M. & Shlomchik M. J. Cutting edge: hierarchy of maturity of murine memory B cell subsets. J. Immunol. 185, 7146–7150 (2010). 21078902 10.4049/jimmunol.1002163 PMC3133669

[b35] Shahaf G. . Antigen-driven selection in germinal centers as reflected by the shape characteristics of immunoglobulin gene lineage trees: a large-scale simulation study. J. Theor. Biol. 255, 210–222 (2008). 18786548 10.1016/j.jtbi.2008.08.005

[b36] Tas J. M. J. . Visualizing antibody affinity maturation in germinal centers. Science 351, 1048–1054 (2016). 26912368 10.1126/science.aad3439 PMC4938154

[b37] Weisel F. J., Zuccarino-Catania G. V., Chikina M. & Shlomchik M. J. A temporal switch in the germinal center determines differential output of memory B and plasma cells. Immunity 44, 116–130 (2016). 26795247 10.1016/j.immuni.2015.12.004 PMC4724390

[b38] Cervenak L., Magyar A., Boja R. & Laszlo G. Differential expression of GL7 activation antigen on bone marrow B cell subpopulations and peripheral B cells. Immunol. Lett. 78, 89–96 (2001). 11672592 10.1016/s0165-2478(01)00239-5

[b39] Toyama H. . Memory B cells without somatic hypermutation are generated from Bcl6-deficient B cells. Immunity 17, 329–339 (2002). 12354385 10.1016/s1074-7613(02)00387-4

[b40] McHeyzer-Williams L. J., Milpied P. J., Okitsu S. L. & McHeyzer-Williams M. G. Class-switched memory B cells remodel BCRs within secondary germinal centers. Nat. Immunol. 16, 296–305 (2015). 25642821 10.1038/ni.3095 PMC4333102

[b41] Berkowska M. A. . Human memory B cells originate from three distinct germinal center-dependent and -independent maturation pathways. Blood 118, 2150–2158 (2011). 21690558 10.1182/blood-2011-04-345579 PMC3342861

[b42] Bemark M. Translating transitions—how to decipher peripheral human B cell development. J. Biomed. Res. 29, 264–284 (2015). 26243514 10.7555/JBR.29.20150035 PMC4547376

[b43] Mora J. R. & von Andrian U. H. Role of retinoic acid in the imprinting of gut-homing IgA-secreting cells. Semin. Immunol. 21, 28–35 (2009). 18804386 10.1016/j.smim.2008.08.002 PMC2663412

[b44] Clemens J. D. . Cross-protection by B subunit-whole cell cholera vaccine against diarrhea associated with heat-labile toxin-producing enterotoxigenic Escherichia coli: results of a large-scale field trial. J. Infect. Dis. 158, 372–377 (1988). 3042876 10.1093/infdis/158.2.372

[b45] Schwickert T. A. . *In vivo* imaging of germinal centres reveals a dynamic open structure. Nature 446, 83–87 (2007). 17268470 10.1038/nature05573

[b46] Staden R., Beal K. F. & Bonfield J. K. The Staden package, 1998. Methods Mol. Biol. 132, 115–130 (2000). 10547834 10.1385/1-59259-192-2:115

[b47] Alamyar E., Giudicelli V., Li S., Duroux P. & Lefranc M.-P. IMGT/HighV-QUEST: the IMGT web portal for immunoglobulin (IG) or antibody and t cell receptor (TR) analysis from NGS high throughput and deep sequencing. Immunome. Res. 8, 26 (2012).

[b48] Barak M., Zuckerman N. S., Edelman H., Unger R. & Mehr R. IgTree: creating Immunoglobulin variable region gene lineage trees. J. Immunol. Methods 338, 67–74 (2008). 18706908 10.1016/j.jim.2008.06.006

