# Supplementary material for: Limited clonal relatedness between gut IgA plasma cells and memory B cells after oral immunization
Source: Nat Commun. 2016 Sep 6;7:12698. doi: 10.1038/ncomms12698 (PMC5025876; doi:10.1038/ncomms12698)
Supplement: Supplementary Information — Supplementary Figures 1-9 [file ncomms12698-s1.pdf]

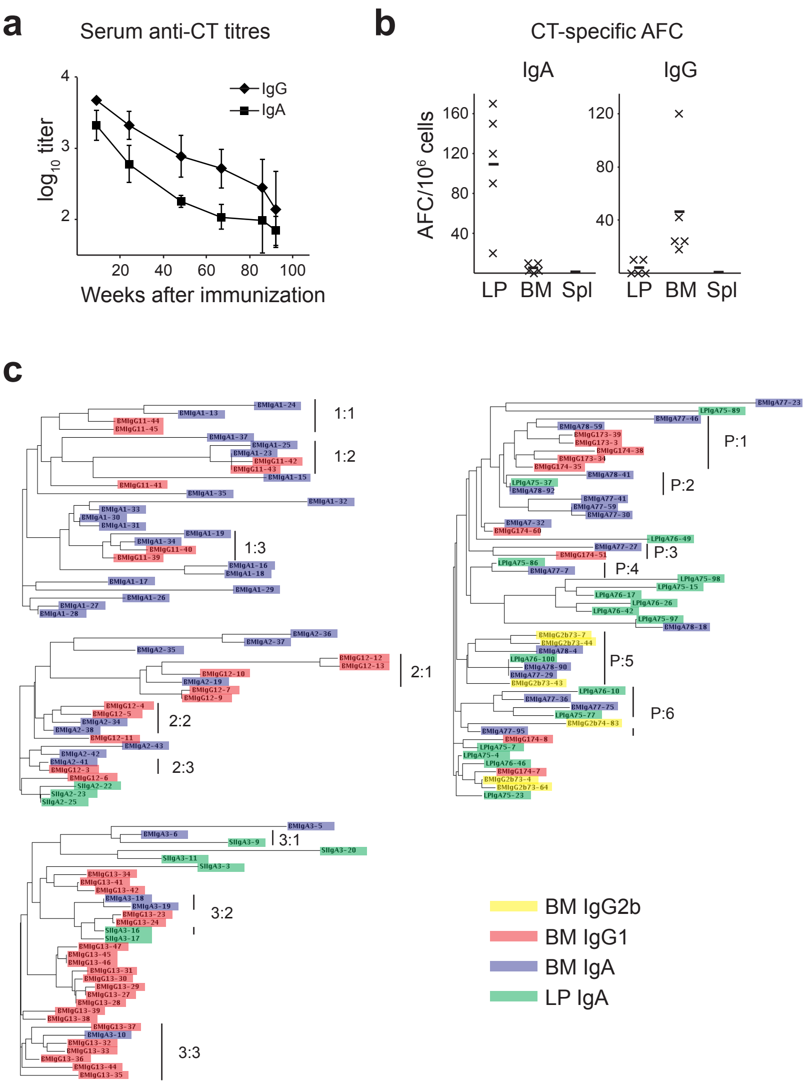

Supplementary Fig. 1. Oral immunization with NP-CT generates long-lived plasma cells.
(a,b) Wild type C57BL/6 mice were orally immunized with NP-CT as in Fig. 1 and (a) the titres of antibodies against CT (IgG = diamonds and IgA = squares with error bars indicating s.d.) were analyzed for 90 weeks and (b) the number of CT-specific AFC were identified in lamina propria (LP), bone marrow (BM) and spleen (Spl) in mice immunized 1 year before analysis. (c) A Clustal Omega analysis was performed on sequences from three mice to identify sequence similarities. Analysis of three individual mice are shown to the left and to a pool of three distinct mice to the right. Clones that share CDR3 VDJ rearrangements are marked with black lines; the CDR3 region and clonal mutations of these clones are shown in Supplementary Fig. 2.

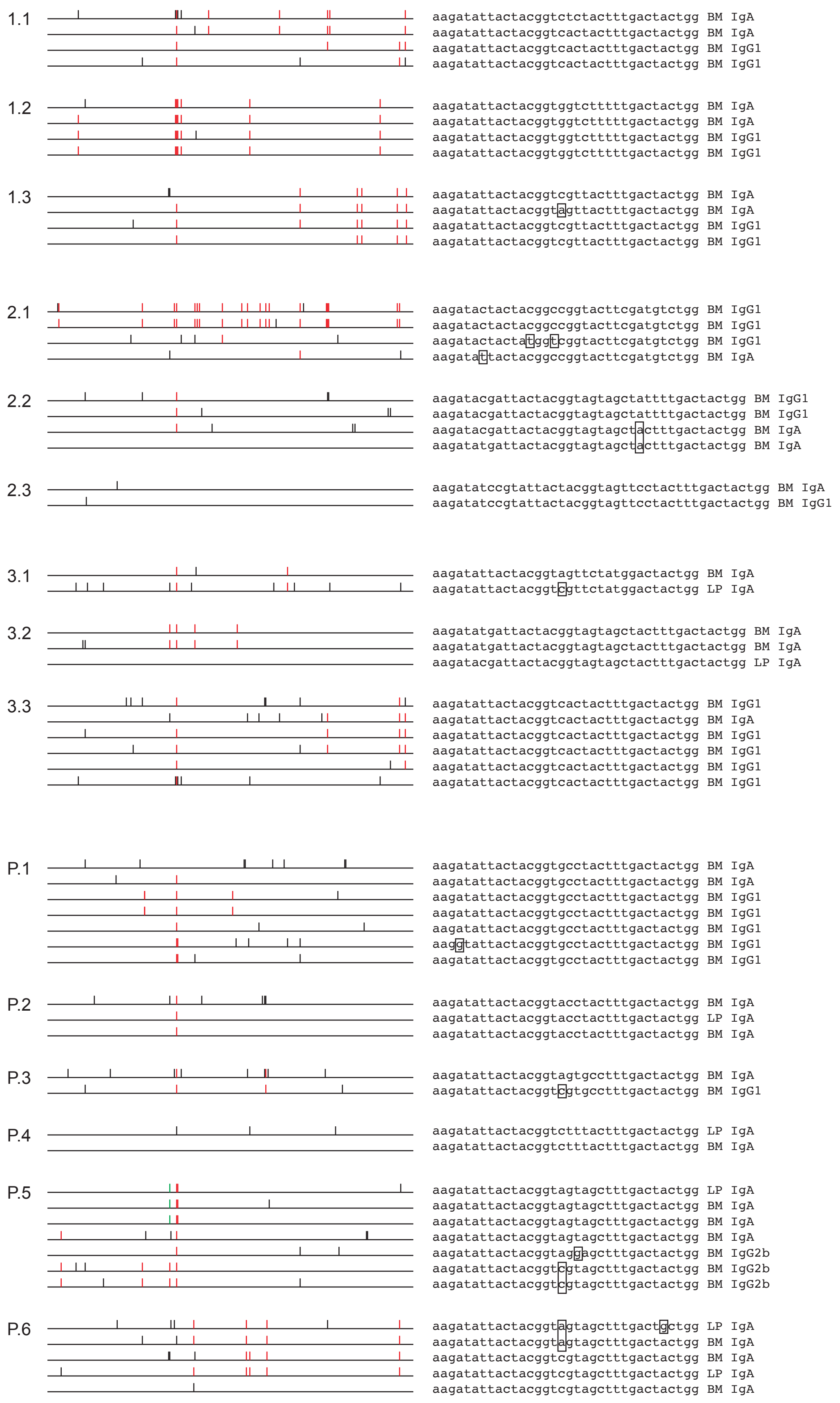

Supplementary Fig. 2. Plasma cell clones in mice one year after oral immunization

Plasma cells with related CDR3 regions were identified by using Clustal Omega analysis as indicated in Supplementary Fig. 1. Mutations within VH186.2 V regions and the sequence of the CDR3 region are indicated for clonally related sequences. Mutations in the V regions that are shared between multiple sequences are shown in red and non-shared mutations in black. Mutations in the CDR3 regions are boxed.

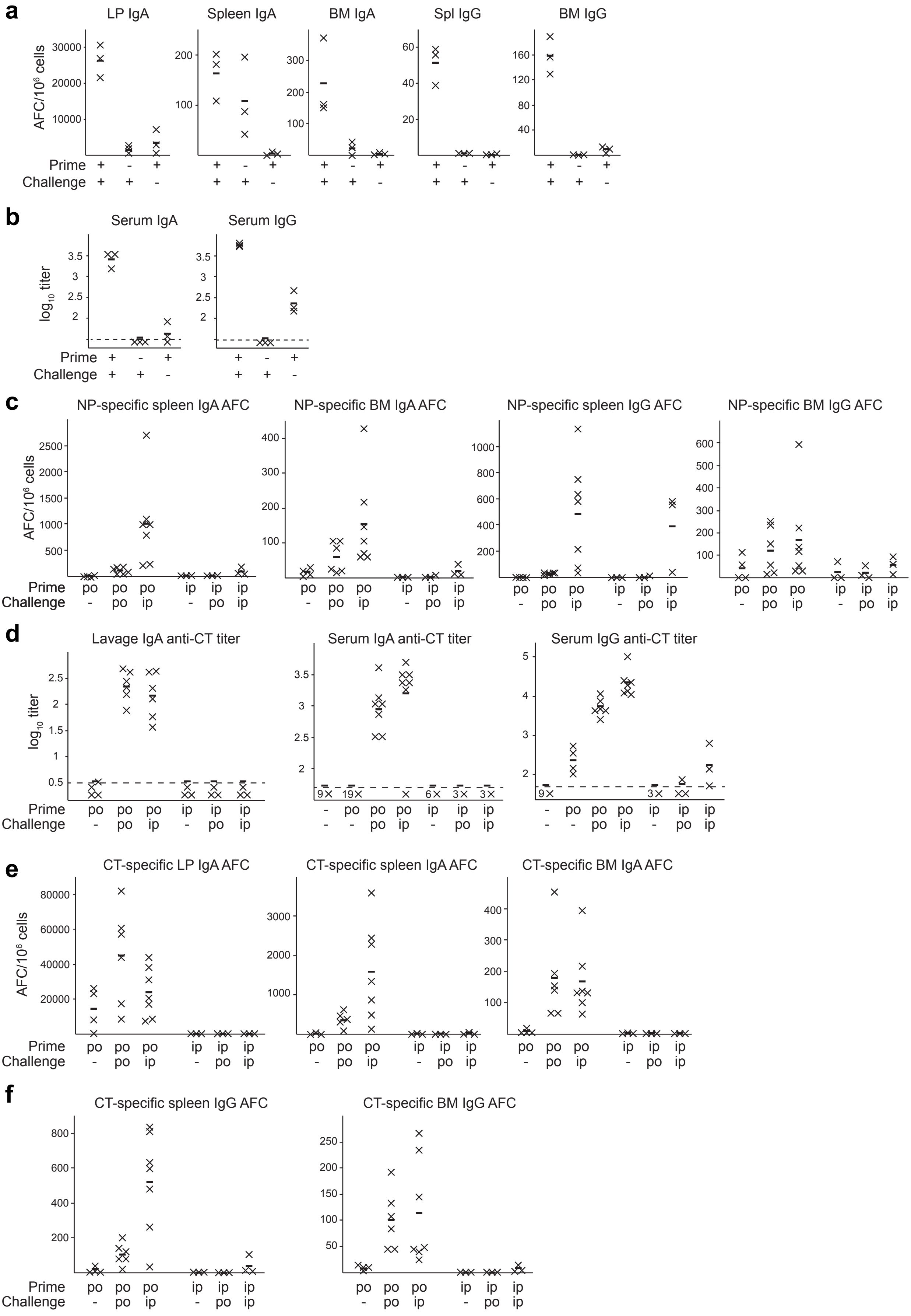

Supplementary Fig. 3. Memory responses after an antigenic challenge one year after immunization. (a,b) The response to CT was determined in orally immunized mice after an oral boost one year after the primary immunization. For further details and data regarding NP response see Fig. 2 a-c. (c-f) The response to NP (c) and CT (d-f) in mice that have been primed either orally with NP-CT x 3 (p.o. groups) or peritoneally with NP-CGG + CTA1-DD x 1 (i.p. groups) and were then challenged with NP-CT x 1 either orally or peritoneally six months later (for more information see Fig. 2h-l). Note that the i.p. primed group was not immunized with the whole toxin, which explains the relatively weak CT responses.

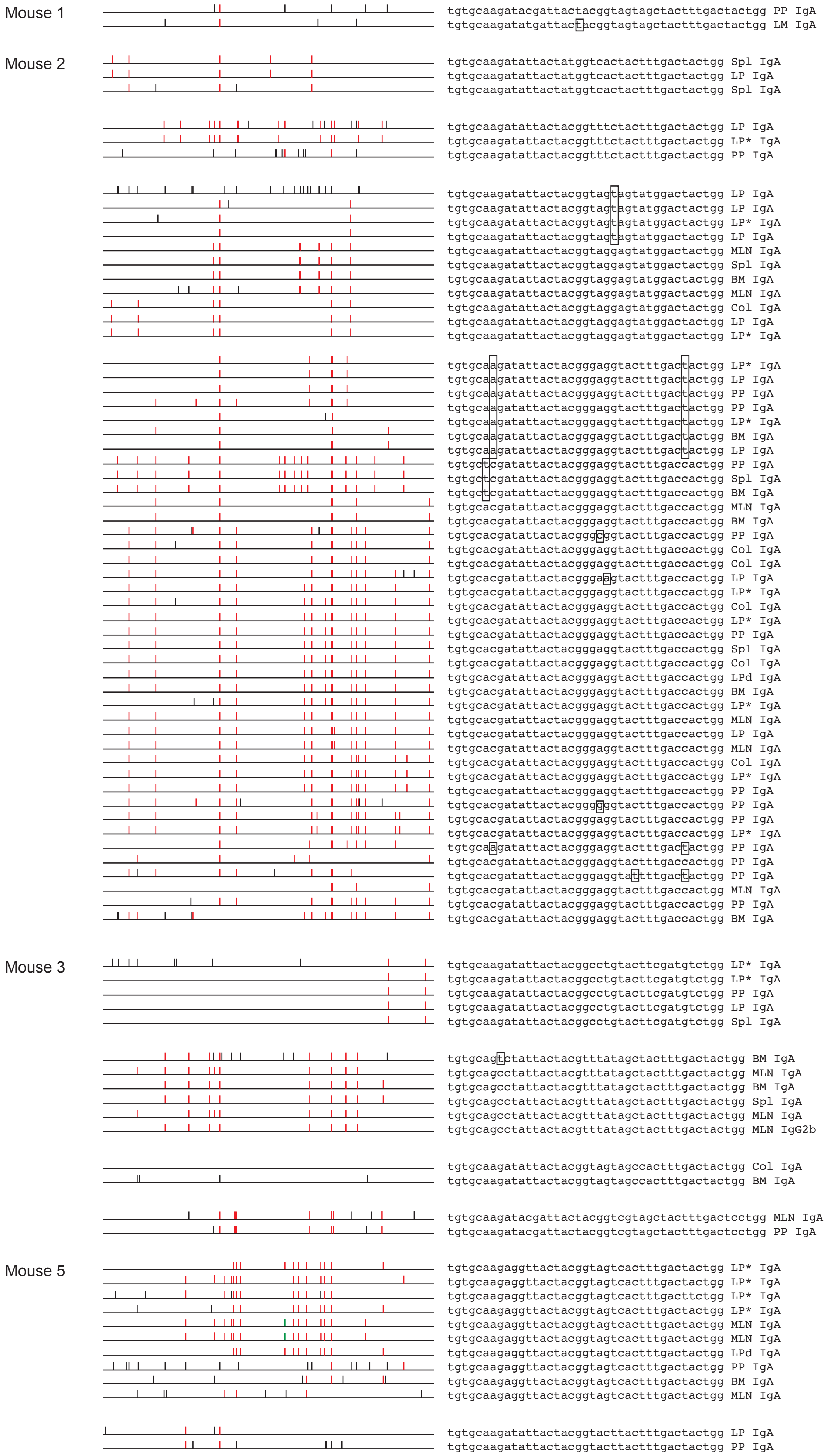

Supplementary Fig. 4. Clonally related sequences detected in mice after an oral challenge.
Clonally related sequences were grouped from mice that had been challenged one year after the primary immunization. Sequences that were not related to any other sequences are not shown. All sequences were used for the analysis shown in Fig. 2e,f.

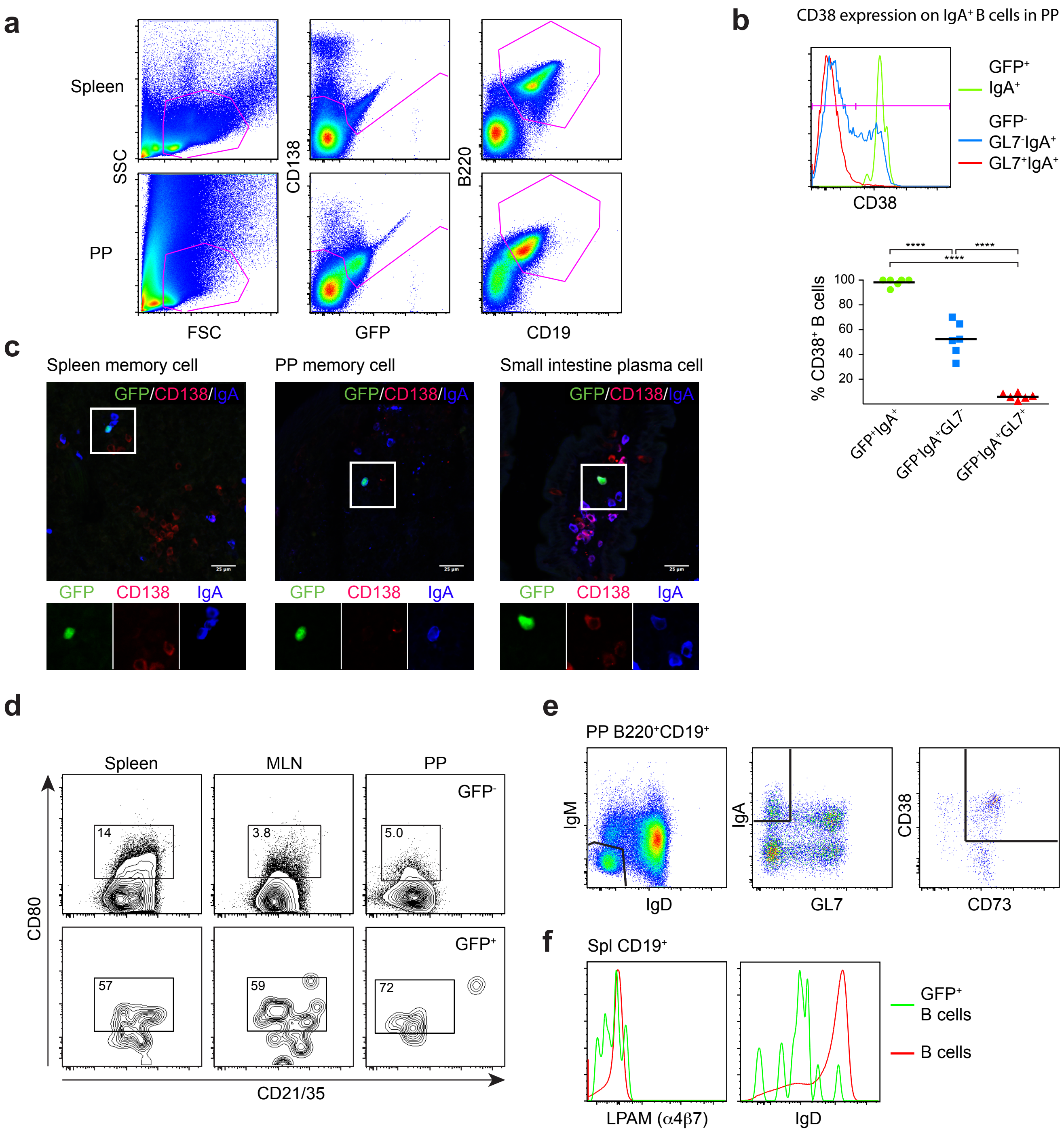

Supplementary Fig. 5. Flow cytometric and microscopic analysis of memory B cells.
(a) Gating strategy used for identifying B cells for further analysis in Fig 3c-f,h,i. Living lymphocytes were gated in FSC/SSC, CD138 expressing cells excluded and $CD19^{+}B220^{+}$ cells gated. (b) $CD19^{+}B220^{+}IgM^{-}IgD^{-}$ cells from PP were analyzed for expression of CD38 on memory $GFP^{+}IgA^{+}$ cells compared to $GFP^{-}IgA^{+}$ cells from that same mouse that were $GL7^{+}$ or $GL7^{-}$. The gate used to determine if cells were $CD38^{+}$ or $CD38^{-}$ is shown on the flow histogram, and the number of cells expressing CD38 in 6 memory mice analyzed after 3-6 months after 3 oral immunizations with NP-CT (lower panel, **** indicates $p<0.0001$). Mice from three independent experiments are shown, with one, two and three mice from each experiment, respectively. (c) Analysis of long-lived memory B cells identified in spleen and PP 6 months after oral immunization with NP-CT using separate channels to determine that IgA memory B cells determined that they were $CD138^{-}$. In contrast, long-lived plasma cells in small intestinal tissues expressed IgA and CD138. The immune fluorescent images of memory cells are representative for five sections with a single memory cell on each section. (d) The expression of CD80 and CD21/35 was determined on NP-binding GFP^{+} B cells and GFP^{-} B cells in mice that had been orally immunized one year before analysis. The number of B cells that expressed CD80 is shown in Fig. 3i. (e) Gating strategy to determine the number of IgA-expressing memory cells in PP, MLN and spleen in aged mice in Fig 3j. B cells that were $IgM^{-}IgD^{-}IgA^{+}GL7^{-}CD38^{+}CD73^{+}$ were identified using the indicated gates on $B220^{+}CD19^{+}$ B cells, and the number of events in the last gate was subsequently divided by the total number of $B220^{+}CD19^{+}$ cells to determine the proportion of IgA-expressing cells with a memory phenotype. (f) GFP-expressing memory B cells were analyzed in mice that had been intraperitoneally immunized with NP-CT 9 months before analysis as described in Fig. 3k. The histogram shows the expression of LPAM (α4β7) and IgD on normal B cells and GFP^{+} memory B cells. The memory B cells also expressed CD73 and PD-L2 (as shown in Fig 3h-i).

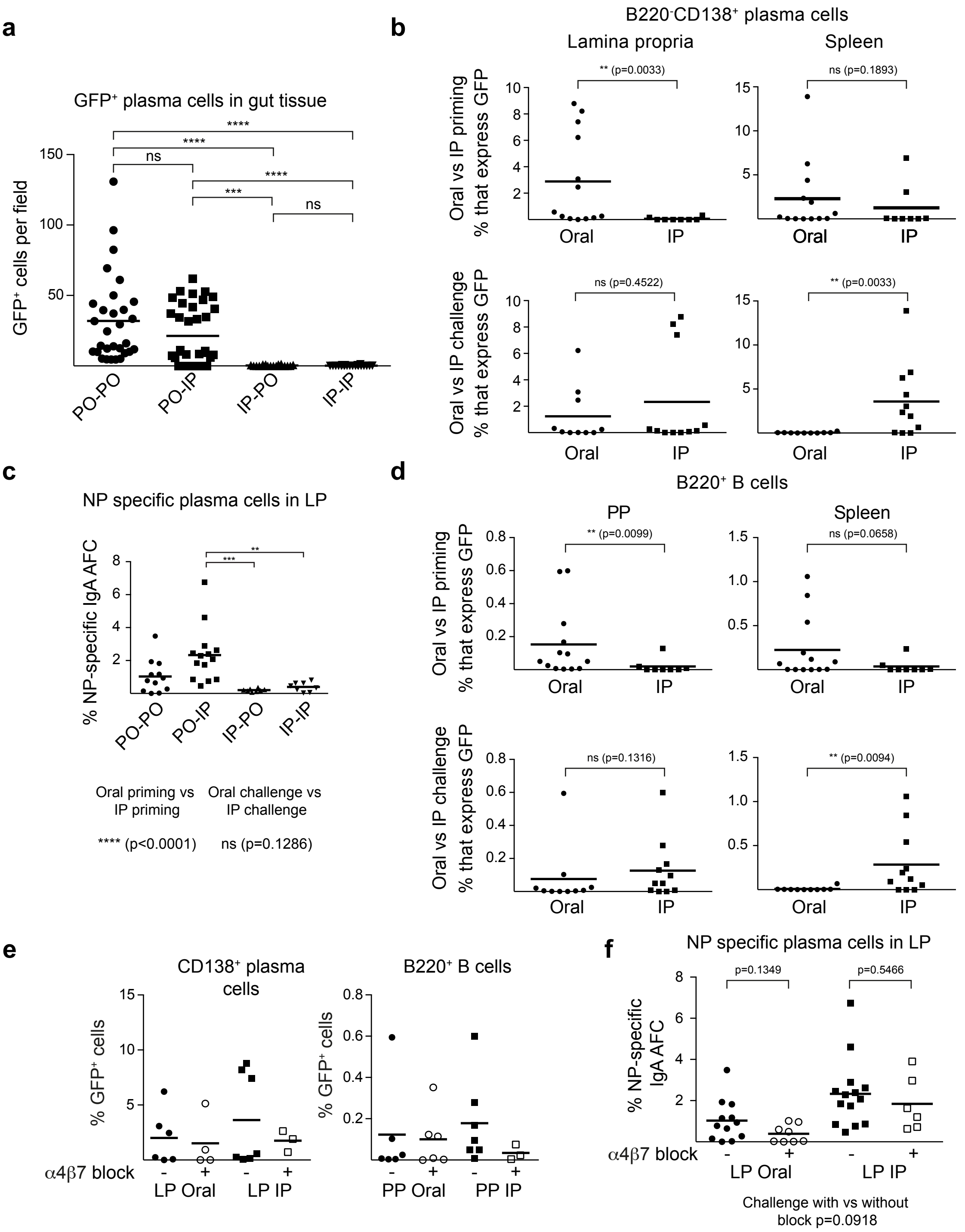

Supplementary Fig. 6. The effect of immunizing mice via the oral or IP route

(a) The number of GFP$^+$ cells per view-field in small intestinal sections was determined using fluorescent microscopy in mice that had been orally (PO) or intraperitoneally (IP) primed with NP-CT 6 months before they were challenged PO or IP using the same antigen as indicated in Fig. 4e. From each section, at least 5 view-fields were counted and the mean of each section is indicated in the diagram. From each mouse, 5 sections were prepared, and each group is based on at least 3 animals. Significance was calculated using the nonparametric Kruskal-Wallis test followed by Dunn´s multiple correction (**** $p<0.0001$, *** $0.0001 \leq p<0.001$). (b,c,d) Statistical analysis of flow cytometric and antigen-specific spot data presented in Fig. 4f-h to determine the number of CD138+ plasma cells (b) and B cells (d) that express GFP or NP-specific antibody forming cells (AFC) (c). For each data set in (b) and (d) data were pooled from mice that had been orally (Oral) or peritoneally (IP) primed, or data from mice that had been orally (Oral) or peritoneally (IP) challenged and the pools were compared using the Mann-Whitney nonparametric test. The data are presented in Fig 4h. In (c) all the four groups were either compared using Kruskal-Wallis test followed by Dunn´s multiple comparison for individual group, or groups were pooled and compared as in (b) and (d). (e) The proportion of CD138$^+$ plasma cells in lamina propria (LP) and B220$^+$ B cells in Peyer´s patches (PP) that expressed GFP was determined in mice that had been orally primed, and orally or IP challenged six months later. The mean number of GFP expressing cells decreased in all groups after blocking with the integrin-α4 β7 antibody DATK32 but the changes were not significant. (f) The numbers of NP-specific IgA cells in LP preparations were compared between mice that were blocked or not with the integrin-α4β7 antibody DATK32 during challenge. Mann-Whitney´s nonparametric test between groups did not reach significance with the indicated p values.

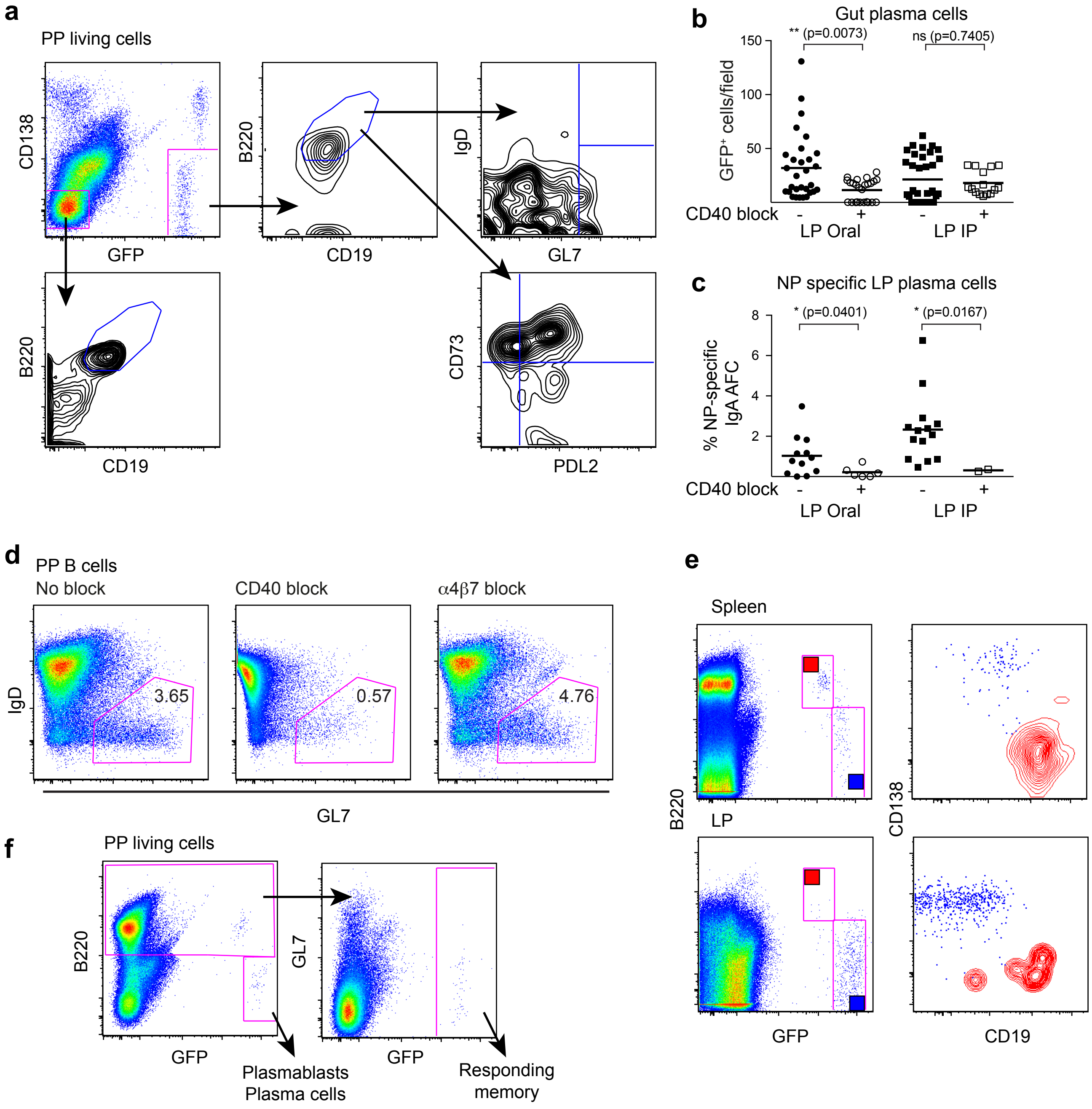

Supplementary Fig. 7. Analysis of responding memory cells in PP and spleen
(a) Gating strategy to identify responding memory cells after booster immunization in Fig. 5a-c,f,g. Lymphocytes were gated as in Supplementary Fig. 5a and from these CD138$^-$GFP$^+$ and CD138$^-$GFP$^-$ cells were gated. A gate for CD19$^+$B220$^+$ B cells was set based on CD138$^-$GFP$^-$ cells and subsequently used for GFP$^+$ cells. The cells were analyzed as indicated in the different panels in Fig. 5. (b) Mice that had been orally primed with three doses of NP-CT were challenged with the same antigen orally (Oral) or intraperiteoneally (IP). The number of GFP expressing plasma cells were then counted in gut lamina propria (LP) sections after CD40 signal blocking using the CD40L blocking antibody MR-1 as described in Supplementary Fig. 6a. The nonparametric Mann-Whitney test was used to analyze the differences between the groups. (c) The proportions of NP-specific IgA cells were compared using ELISPOT between mice that had been CD40L blocked or not. The nonparametric Mann-Whitney test was used to analyze the differences between the groups. (d) Flow cytometric analysis of B220$^+$CD19$^+$ B cells demonstrated that CD40 blocking using anti-CD40L MR-1 disrupted germinal center reactions in PP whereas the integrin-α4β7 blocking antibody DATK32 did not. (e) NP-specific GFP$^+$ plasma cells/plasmablasts can be distinguished based on B220 and GFP expression levels. Two expanded GFP$^+$ populations, comprised of B220$^-$GFP^{high} and B220$^+$GFP^{int} cells, were evident in spleen after i.p. immunization whereas B220$^-$GFP^{high} cells dominated in extracts from small intestinal lamina propria. B220$^+$GFP^{int} cells were exclusively CD138$^-$CD19$^+$ expanding memory cells and B220$^-$GFP^{high} were CD138$^+$CD19$^{-/low}$ plasma cells/plasmablasts. (f) Gating of B220 and GFP as indicated in (e) allowed us to separately analyze the expression of IgA, CCR9, CCR10 and α4β7 on responding memory cells and plasma cells/plasmablasts in Fig 5i,j.

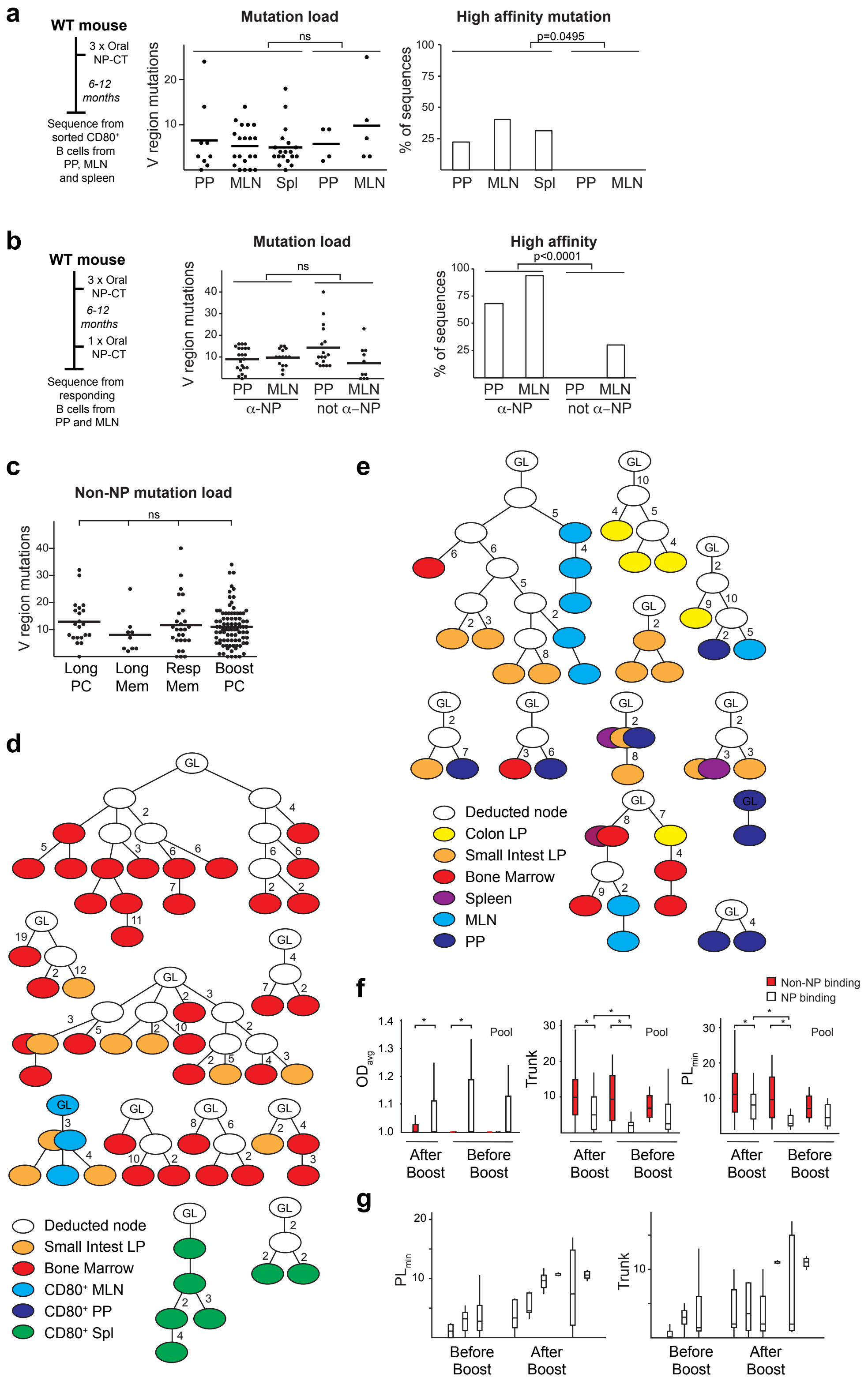

Supplementary Fig. 8. Comparison between memory and challenge responses.
(a and b) The number of V region mutions and the number of cells carrying high affinity mutations among IgA-switched CD80^{+} memory cells (a) or cells responding to a challenge (b). (c) The number of mutations in non-NP binding VH186.2 V regions isolated from mice before (Long PC and Long Mem) and after (Resp Mem and Boost PC) a challenge immunization. There were slightly fewer mutations in the Long Mem group than the others, but the difference was not significant. (d and e) Trees constructed from sequences in non-boosted (d) and boosted (e) mice (two additional trees from each can be found in Fig. 6 d, e). All trees with two sequences or more are shown. Only a single tree out of 12 shared sequences between memory and long-lived plasma cells (d) whereas 8 out of 13 trees shared sequences between responding memory cells (in MLN and PP) and plasma cells formed after boosting (BM, LP and Spl). (f and g) Tree shape statistics derived from tree analysis. (f) Trees formed from non-NP bindings sequences (red) are compared to trees formed from NP-binding sequences (white) in the same animal (* $p<0.05$). An analysis of sequences derived from a pooled group of three before boost animals are also shown. As this group is not fully comparable to the single mice groups statistics are not given, but the results resembled the other before boost groups. (g) Tree statistics from individual mice that are analyzed in groups in Fig. 6 f. The medians for all mice (n=3) in the before boost group were lower than in any of the mice in the after boost (n=6) group for PLmin, which is a significant difference (p=0.0238), whereas one mice in the before boost group over-lapped with mice in the after boost group with regard to trunk length, making it non-significant.

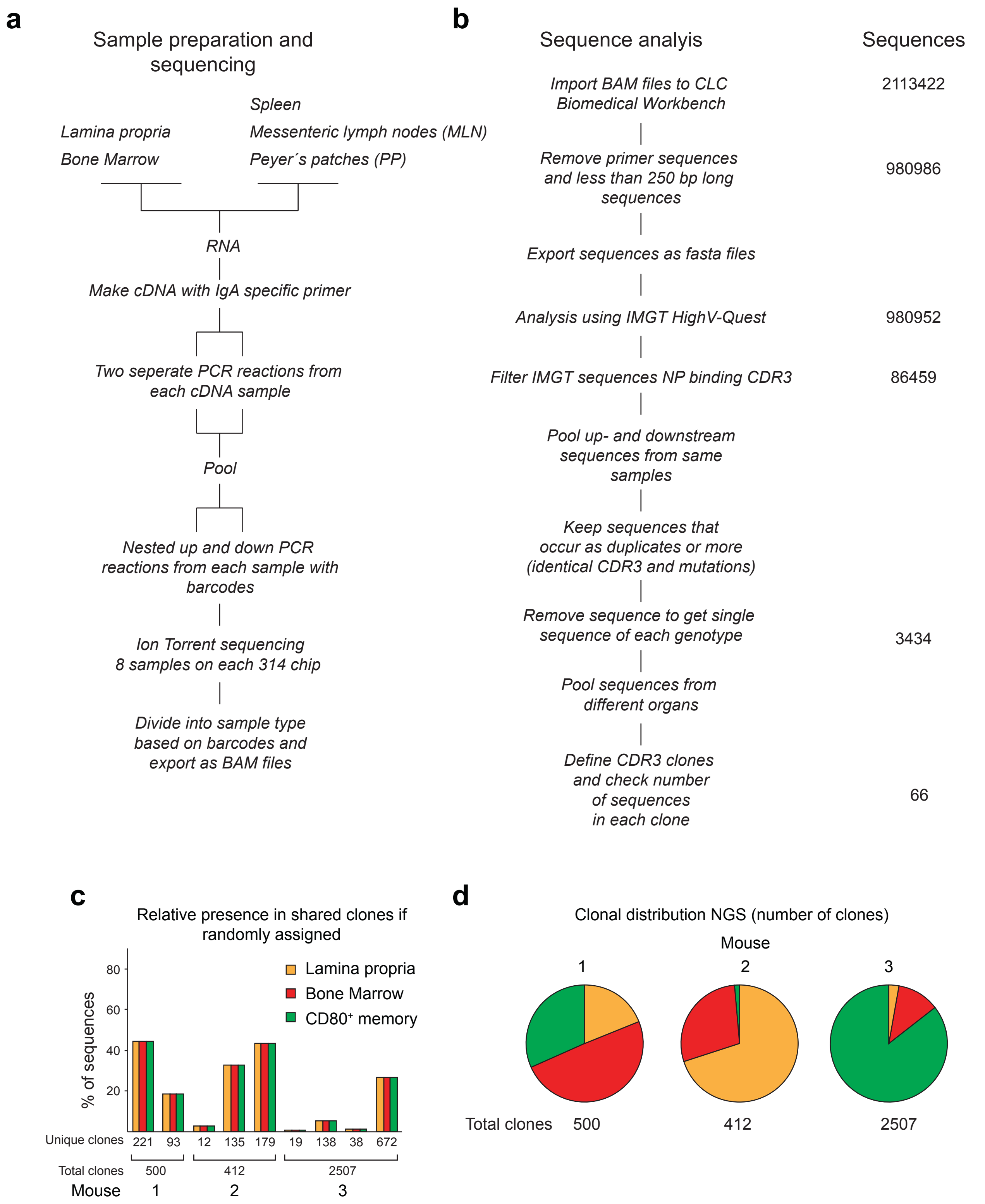

Supplementary Fig. 9. Next generation sequence analysis of antibody genes binding to NP
(a) This show an overview of the steps performed in the laboratory for the sequencing, and (b) the analysis of the sequence files and the number of sequences left at each analysis step. RNA was prepared from gut lamina propria, cells flushed out from the bone marrow or prepared from sorted CD80+ memory B cells from spleen, MLN and PP and cDNA made using an IgA specific primer. The cDNA from each sample was divided into two and PCR amplified in separate reactions as described in Material and Methods section. After pooling and purification of the PCR products, the amplified cDNA was used for a nested PCR using upstream primers aligning to the FRW1 region of the VH186.2 and related V genes and downstream primers aligning in the IgA constant region just downstream of the J regions. Either the downstream primer included the trP1 and the upstream A sequences, or vice verse as described in the Ion Torrent Ion Amplicon Library Preparation (Fusion Method) instruction (Publication Number 4468326, revision C). IonXpress barcodes 001-008 were inserted between the A and primer sequences in the primers to generate four individual PCR reactions for upstream and four for downstream sequencing. After purification of PCR products using a MinElute PCR purification kit (Qiagen), PCR products were confirmed on gel and the

concentration of DNA was determined using a Qubit fluorometer. An IonChef was subsequently used to load six-eight distinct PCR products on each 314 chip, and the chip that was subsequently sequenced using 400 bp HiQ sequencing. The resulting sequences were divided into groups based on barcodes and were exported as BAM files using the Ion Torrent Suite software. In total, 2,113,422 sequences were imported into CLC Biomedical Workbench software version 9.0 (Qiagen) where primer sequences and sequences less than 250 bp long were removed and the resulting 980,986 sequences were exported as fasta files. The fasta files were submitted for IMGT HighV-Quest analysis (www.imgt.org), which resulted in that 980,952 sequences were aligned to antibody genes. NP binding sequences were identified among these based on that they used the VH1-72 V region (i.e. the VH186.2 gene), that they had CDR3 regions between 11-14 amino acids long, that the fourth amino acid in the CDR3 region was a tyrosine and that there were at least two more tyrosines in the following three positions. A total of 86,459 NP binding sequences were identified, and these were screened for numbers of mutations in the CDR1-CDR3 region and the presence of the affinity increasing CDR1 mutation as described in Fig. 6h. To avoid mutations that were due to sequencing errors or misreading of barcodes, upstream and downstream sequences were pooled for each site, and only sequences that had a partner with an identical CDR3 and mutation pattern were kept. Each genotype (i.e. sequences with identical CDR3 and mutation pattern) were exported (i.e. if 10 identical sequences were present, only one copy of the sequences was kept), resulting in 3,434 unique genotypes. All genotypes from each mouse were then pooled, and were aligned into clones based on CDR3 regions, defined as allowing for a distance of three base pair differences in the CDR3 to belong to a clone. In total, 66 clones were identified with between 1 and 863 sequences in each clone. Clones with more than two sequences are shown in Fig. 6j with clone sizes indicated by circle size and the part of the clone made up by plasma cell or memory cell sequences as portions of the circle. In Fig 9i, the relative number of sequences from a site (i.e. lamina propria, bone marrow or CD80+ memory cells) are shown for clones that contained sequences from more than two sites. No memory cells with clonal relationships to plasma cells were found in mouse 1 and 2, while 24 % of memory cell sequences in the third mouse were present in two clones that also included long-lived plasma cells from lamina propria and bone marrow. (c and d) This shows the result if the genotypes were randomly distributed within clones presented to be comparable to that in Fig 6i and j. (c) The number of sequences with unique genotypes that were present in each mouse is indicated, as well as the number of unique sequences that belonged to the clones that are presented in Fig 6i. The relative presence in the respective clone would have been equal for lamina propria, bone marrow and memory had the sequences been randomly distributed. (d) The actual number of sequences with unique genotypes that were made up of lamina propria, bone marrow and memory sequences are illustrated in pie charts. Had there been a random distribution in Fig 6j, each pie would have hade this distribution.
